# Factors influencing the implementation of general practice nurse-delivered models of care for chronic conditions: a mixed-methods systematic review to inform models of care for chronic sleep disorders

**DOI:** 10.1186/s12875-025-03078-4

**Published:** 2026-02-14

**Authors:** Nicole Grivell, Brandon Brown, Jeffrey Fuller, Ching Li Chai-Coetzer, R. Doug McEvoy, Elizabeth Hoon

**Affiliations:** 1 National Centre for Sleep Health Services Research, College of Medicine and Public Health, Flinders Health and Medical Research Institute, Flinders University, Adelaide, SA 5042 Australia; 2 Adelaide Institute for Sleep Health/FHMRI Sleep Health, College of Medicine and Public Health, Flinders Health and Medical Research Institute, Flinders University, Adelaide, SA 5042 Australia; 3 Department of Physiotherapy, Melbourne School of Health Science, University of Melbourne, Melbourne, VIC 3010 Australia; 4 Institute for Breathing and Sleep, Austin Health, Melbourne, VIC 3084 Australia; 5 College of Nursing and Health Sciences, Flinders University, Adelaide, SA 5042 Australia; 6 Department of Respiratory, Sleep Medicine and Ventilation, Southern Adelaide Local Health Network, SA Health, Adelaide, SA 5042 Australia; 7 Discipline of General Practice and Adelaide Rural Clinical School, Faculty of Health Sciences, University of Adelaide, 5000, Adelaide, SA 5000 Australia

**Keywords:** Chronic disease management, Systematic review, General practice, Patient centred care, Nursing, Barriers, Facilitators, Health services research, Implementation research, Models of care

## Abstract

**Background:**

General practice nurse-delivered models of care for obstructive sleep apnoea and chronic insomnia have been investigated in response to long wait times for specialist sleep services and limited access to recommended treatments. Despite evidence of effectiveness, these models have not yet been implemented into routine care. Understanding the factors influencing implementation can facilitate translation of research evidence into practice, however no research exploring the barriers and facilitators to the implementation of general practice-delivered models of care for sleep health was identified. Therefore, a systematic review exploring the barriers and facilitators to the implementation of general practice nurse-delivered models of care for chronic conditions was conducted to inform the implementation of new models of care for chronic sleep disorders.

**Methods:**

A mixed-methods convergent integrated systematic review. Seven databases (MEDLINE (Ovid), CINAHL (EBSCO), Embase, Scopus, Cochrane Library, Emcare (Ovid), and ProQuest Theses and Dissertations) and Google Advanced Search were searched to July 2025, to identify records reporting barriers and/or facilitators to the implementation of nurse delivered care for chronic conditions in the general practice setting. Data were extracted and mapped to a conceptual framework for the implementation of complex interventions in the primary care setting and synthesised using framework synthesis.

**Results:**

Eighty-five records were included in the review. It was identified that general practice staff believed that having a shared understanding about the benefits of the model of care, support from specialist staff, and compatibility of the model of care with existing work processes facilitated implementation. Busy workloads, inadequate funding models, and uncertainty about the scope of practice of nurses were barriers to implementation. Patient-related factors were identified, such as patient capability and willingness to participate, but they did not fit meaningfully into the conceptual framework. This necessitated development of an amended determinant framework for the implementation of general practice-delivered models of care for chronic conditions (Factors influencing the Implementation of models of care for Chronic Conditions in General Practice; FICC-GP).

**Discussion/Conclusion:**

This review provides evidence, recommendations, and a determinant framework to support the implementation of new models of care for chronic conditions in general practice. Greater consideration of patient need and motivations for engagement in chronic condition management, improved clarity about the scope of practice of general practice nurses, and co-design with general practice staff and consumers may offer greater opportunities for success when designing and delivering new models of care for chronic conditions, including chronic sleep disorders.

**Trial registration:**

This review has been registered with PROSPERO (CRD42021273346).

**Supplementary Information:**

The online version contains supplementary material available at 10.1186/s12875-025-03078-4.

## Background

Chronic conditions, which are defined as medical conditions that are long-lasting, have an impact on health, and require medical treatment, are a growing concern due to their negative impacts on health and wellbeing [1, 2]. Chronic conditions are associated with depression and reduced health-related quality of life, and are a leading cause of death worldwide [3–5]. Assessment and management of chronic conditions is frequently conducted in general practice, a primary care setting in which general practitioners (can also be referred to as family or primary care physicians) provide clinical care, often in conjunction with nurses (general practice nurses) [6]. General practice offers a patient-centred approach to care that includes the development of personalised care plans, long-term monitoring of symptoms and functioning, and patient self-management education [7–9]. Widely recognised chronic conditions include chronic obstructive pulmonary disease, diabetes, and chronic heart failure, however there is growing recognition of the chronicity of the two most common sleep disorders, obstructive sleep apnoea and chronic insomnia, and a push to adopt comprehensive chronic condition management strategies to optimise patient outcomes [10–13].

Obstructive sleep apnoea (OSA) is characterised by repeated partial or complete airway obstructions during sleep and can present with symptoms of daytime sleepiness, reduced mood, and poor concentration [14–16]. Recommended first line treatment for symptomatic OSA is continuous positive airway pressure (CPAP), positive airway pressure applied to the upper airway at night using a mask to splint the airway open [17]. However, CPAP is not a cure for OSA, and the benefits of CPAP rely on patient adherence to therapy, which can be sub-optimal due to discomfort and/or intolerance [18]. Best practice management of OSA involves ongoing monitoring of symptoms, side effects, and treatment response, even if there is good adherence to CPAP therapy [19]. Similarly, treatments are available for chronic insomnia, which is defined as difficulty getting to sleep, staying asleep, or waking too early with daytime symptoms for at least three months [20]. Of note, patient engagement and ongoing adherence to treatment recommendations are also required to achieve optimal outcomes. First line recommended treatment for chronic insomnia is cognitive behavioural therapy for insomnia (CBTi), an intensive six-to-eight-week treatment program delivered by a trained clinician, followed by ongoing monitoring for reoccurrence of symptoms [21, 22]. Following completion of CBTi, patients are expected to monitor for the recurrence of symptoms of insomnia and apply the cognitive and behavioural principles that they learnt during the treatment program. While 70–80% of patients have a therapeutic response to CBTi, as many as half of patients treated with CBTi have recurrence of their insomnia symptoms making chronic insomnia, like OSA, a largely incurable but manageable chronic condition [23, 24].

In recent years there has been a growing interest in the development of general practice-delivered models of care for the assessment and management of OSA and chronic insomnia [25–29]. This interest has been prompted by an increased recognition of barriers to accessing sleep health care. Long wait times for publicly-funded OSA assessment and management, insufficient trained providers of CBTi, and inequities in access to sleep health care for individuals living in rural and remote areas and/or on low income, make accessing assessment and treatment of OSA and chronic insomnia a challenge for some individuals [30–35]. In an effort to improve access to sleep health care, randomised-controlled trials have tested new models of care for the assessment and management of OSA and chronic insomnia delivered in primary care by general practice nurses [36–41]. These models of care offer treatment delivered by familiar health care providers in local communities at a reduced cost and have been shown to be effective, however they have not yet been implemented into routine care.

To transition nurse-delivered models of care from research into clinical practice it is important to look beyond randomised controlled trials and consider the contextual factors that could influence implementation and care delivery [42]. Applying this knowledge to the design and implementation of interventions has been shown to facilitate the translation of research findings into practice, with the goal being to bridge the commonly reported 17-year gap between research and practice [42–44].

Research exploring the implementation of nurse-delivered models of care for OSA and chronic insomnia is scarce [45], however general practice nurses have had an enhanced role in the assessment and management of other chronic conditions for more than 20 years [46, 47]. Over this time, the role of the general practice nurse has expanded and a role for nurses in the delivery of models of care for chronic conditions have been explored for many other chronic conditions, such as diabetes and cardiovascular disease [48–50]. With years of research exploring the implementation of general practice nurse-delivered models of care for these conditions, it seemed opportune to use the learnings from these models of care to inform the design and implementation of new models of care for OSA and chronic insomnia. This systematic review was conducted to answer the research question: “What barriers and facilitators influence the successful implementation of nurse-delivered models of chronic disease management within general practice?”.

## Methods

A Joanna Briggs Institute (JBI) mixed methods convergent integrated systematic review was conducted, informed by the PRISMA 2020 checklist [51, 52]. Data extraction and synthesis was conducted using the principles of ‘best fit’ framework synthesis [52]. A framework synthesis was conducted as it provides structure to large and complex reviews, facilitates exploration of connections and meaning in the data, and produces data that can be used pragmatically within clinical practice [53, 54]. A ‘best fit’ approach was used as a relevant framework, a conceptual model of factors influencing the implementation of complex interventions into primary care (referred to in this paper as the “Lau framework”), was available [55, 56]. The findings were also interpreted in relation to the Chronic Care Model (CCM), a widely accepted best practice approach to chronic condition management that defines six essential elements to the successful delivery of care for chronic conditions: self-management support, decision support, health care organisation, community resources, clinical information systems, and delivery system redesign [57, 58]. A comprehensive search of systematic reviews was conducted and no previous review on this topic was identified. Further detail about the methods employed in this review can be found in the published protocol paper [59].

### Eligibility criteria

Eligibility criteria for this review were defined using the PICo framework for qualitative research questions (*P*articipant, phenomenon of *I*nterest, and *Co*ntext) [52, 60]. The inclusion and exclusion criteria are listed in Table 1.

### Participants

Reports exploring the implementation of models of care for the assessment and management of chronic conditions for adults (individuals aged ≥ 18 years) were considered for inclusion. The priority chronic conditions of the World Health Organization were chosen as the chronic conditions included in this review: cardiovascular diseases, cancer, chronic respiratory diseases, and diabetes [61], along with OSA and insomnia to ensure that any data pertaining to these specific chronic conditions of interest were identified.

### Phenomenon of interest

Records that included any data pertaining to barriers or facilitators to the implementation of nurse-delivered models of care for the included chronic conditions were considered for inclusion. The terms “barriers” and “facilitators” were used in this review as they are accepted terms to describe cause and effect in primary care research [56].

### Context

Studies that were conducted in the general practice setting in countries with a developed economy (as defined by the United Nations) were considered for inclusion [62]. Countries with a developed economy were chosen as the context for this review as these countries have comparable health care systems to Australia, the country in which implementation efforts informed by the findings of this review are expected to occur.

**Table 1:** Inclusion and exclusion criteria

Inclusion Criteria	Exclusion Criteria
Nurse-delivered or nurse-led intervention studied	Not a nurse-delivered or nurse-led intervention studied
Intervention delivered in the general practice (family medicine) context	Intervention not delivered in the general practice (family medicine) context (primary care settings that are not general practice/family medicine are excluded)
Includes data about barriers, facilitators or factors influencing the implementation of the intervention	Does not refer to barriers, facilitators or factors influencing implementation
Relating to the assessment and management of the following conditions: • chronic respiratory diseases • cancer • cardiovascular diseases • diabetes • OSA • insomnia	Intervention is not related to conditions listed in the inclusion criteria
Intervention provided to adults (≥ 18 years)	Intervention is provided to adolescents or paediatrics
Primary qualitative, quantitative or mixed-methods research including theses and dissertations	Systematic reviews or opinion pieces/commentary/letters to the editor
Published in English	Published in a language other than English
Study conducted in a country with a developed economy as defined by the United Nations	Study conducted in any country that is not considered by the United Nations to have a developed economy

### Search strategy

A limited database search was conducted to inform the full search strategy. This was followed by systematic searches of seven databases: MEDLINE (Ovid), CINAHL (EBSCO), Embase, Scopus, Cochrane Library, Emcare (Ovid), and ProQuest Theses and Dissertations to July 4th, 2025. No start date for the search was set so that all literature relevant to the review could be identified. The full search strategies are available in Additional file 1. The reference lists of included records were screened for relevant records, and a Google Advanced search (up to July 4th, 2025; logged out using incognito mode) was conducted to identify any additional literature.

### Study selection and critical appraisal

All records were uploaded into EndNote v.20 (Clarivate Analytics, PA, USA) and duplicates were manually removed. Unique records were then uploaded to Covidence software (Veritas Health Innovation, Melbourne, Australia) for independent title and abstract screening by reviewers 1 and 2. All records that met the inclusion criteria then progressed to independent full text screening by two reviewers (reviewer 1 and either reviewer 2 or 3). If additional information was required for decision making about suitability for inclusion, attempts were made to contact the corresponding author of the record. Any discrepancies in decisions were resolved by discussion between the reviewers. Consensus was reached for all decisions. JBI tools were used independently by reviewers 1 and 2 to quality appraise all included records [63–67].

### Data extraction and synthesis

A data extraction form was used (see protocol paper [59]) and characteristics of included records are listed in Table 2. Data extraction was conducted by reviewer 1 and collated in a data extraction table (available in Additional file 2). Reviewers 2 and 3 checked the data extraction of a total of 20% of included records, and findings were discussed. These discussions led to deeper consideration of the meaning of the data and informed subsequent data extraction and coding of the full dataset, which increased rigour of the findings. Data were then backwards mapped into the primary and secondary themes of the Lau framework, and an additional primary theme was created (the evolution of this theme and an amended determinant framework is described in results), forming a data synthesis table which is presented as Table 3 [56]. Further analysis of the data within secondary themes was conducted using thematic analysis to derive greater meaning from the mapped data [149]. These derived themes are marked in italics and parentheses in Table 3. The year of publication, chronic condition studied, and country of origin of the record were considered during data synthesis and analysis.

**Table 2:** Included records characteristics table

First author and year of publication	Name of conditions studied	Country of origin	Study design	Health professionals delivering the study intervention	Classification of nurses delivering the intervention	Population of interest	Description of the intervention
Hoskins, 2016 [68]	Asthma	UK	Mixed methods	Practice asthma nurse	Classification not specified	Adults with asthma	Goal setting and the development of an action plan with patients
Jones, 1995 [69]	Asthma	UK	Randomised controlled trial	Practice nurses	Classification not specified	Patients with asthma	Nurse-led proactive asthma clinic
Morrow, 2017 [70]	Asthma	UK	Qualitative research	Not applicable – exploratory qualitative study	Not applicable	Children over 5 and adults	Not applicable - an exploration of GP, practice nurse and administrative staff perspectives towards nurse-delivered asthma management
Pinnock, 2005 [71]	Asthma	UK	Qualitative research	Practice nurses	Classification not specified	Patients with asthma	Telephone and face to face-delivered consultations for asthma reviews
Rupasinghe, 2021 [72]	Asthma	Australia	Qualitative research	Not applicable – exploratory qualitative study	Not applicable	Patients with asthma	Not applicable – an exploration of GP and practice nurses’ current management of asthma
Upton, 2011 [73]	Asthma	UK	Qualitative research	Not applicable – exploratory qualitative study	Not applicable	Patients with asthma selecting an inhaler	Not applicable - exploratory qualitative study of the perspectives of registered nurse/practice nurses who were attending a respiratory course training day towards nurse-delivered asthma care
Van Gaalen, 2016 [74]	Asthma	The Netherlands	Qualitative research	GPs and practice nurses	Classification not specified	Adult patients with asthma	Internet-based self-management support
Wiener-Ogilvie, 2008 [75]	Asthma	UK	Qualitative research	Practice nurses and GPs	Classification not specified	Patients with asthma	Exploration of barriers and facilitators to the implementation of an asthma guideline
Zwar, 2022 [76]	Asthma	Australia	Cohort study	Practice nurses	Classification not specified	Patients with asthma	GASP program of patient assessment and education for asthma
Perry, 2008 [77]	Cancer	UK	Qualitative research	Practice nurses	Primary care cancer nurse - classification not specified	Patients with cancer	Integration of a primary care cancer nurse into the general practice setting
Halcomb, 2007 [78]	Cardiovascular disease	Australia	Qualitative research	Not applicable – exploratory qualitative study	Not applicable	Not applicable	Not applicable – an exploration of the perspective towards medical and nursing experts participating at a conference towards the barriers to the implementation of an advanced role for practice nurses within cardiovascular disease management
Halcomb, 2008 [79]	Cardiovascular disease	Australia	Mixed methods research	Not applicable – exploratory qualitative study	Not applicable	Exploratory study about the development of the role of the practice nurse within cardiovascular disease management	Not applicable – an exploration of practice nurse perspectives towards the barriers and facilitators to the development of the role of the practice nurse within cardiovascular disease management
Angus, 2012 [80]	COPD	UK	Quasi-experimental - no control	Practice nurses	Classification not specified	Patients with COPD	Computer-guided nursing consultation using the NICE guidelines
Ansari, 2018 [81]	COPD	Australia	Process evaluation	Practice nurses and GPs	Classification not specified	Patients with newly diagnosed COPD and other co-morbidities	Tailored self-management education program
Faulkner, 2016 [82]	COPD	UK	Mixed methods research	Practice nurses	Classification not specified	Patients receiving spirometry for assessment of COPD	Delivery of spirometry and review of report
Kolltveit, 2023 [83]	COPD	Norway	Qualitative research	Practice nurses and GPs	Classification not specified	Patients with COPD or current smokers at risk of COPD	Guided Self‑Determination counselling program for to improve self-management of COPD
Lofdahl, 2010 [84]	COPD	Sweden	Cross sectional study	Not applicable – an exploratory study	Classification not specified	Patients with COPD	Not applicable – a retrospective exploration of nurse-delivered COPD management
Molina-Vázquez, 2025 [85]	COPD	Spain	Cyclic implementation project	Primary care nurses	Classification not specified	Patients with COPD	Delivery of best practice care for COPD following discharge from hospital
Voncken-Brewster, 2014 [86]	COPD	The Netherlands	Mixed methods research	Practice nurses	Classification not specified	Patients with COPD	Online behaviour change application
Walters, 2012 [87]	COPD	Australia	Mixed methods research	Practice nurses	Registered Nurse	Adults with COPD	Health mentoring for COPD self-management by nurses
Weldam, 2017 [88]	COPD	The Netherlands	Randomised controlled trial	Practice nurses	Classification not specified	Adults with COPD	Development of a booklet describing intervention and a short, animated movie for patients
Dontje, 2013 [49]	Coronary artery disease	USA	Cohort study	Nurse practitioners	Nurse Practitioner	Patients with stable coronary artery disease	Nurse-led group education visits
Nurmeksela, 2021 [89]	Coronary artery disease	Finland	Cohort study	Practice nurses	Registered Nurses and Advanced Practice Nurses	Patients with coronary artery disease	Counselling for self-management of coronary artery disease
Murchie, 2005 [90]	Coronary heart disease	UK	Qualitative research	GPs and nurses	Classification not specified	Patients with coronary heart disease	Secondary prevention clinics for coronary heart disease
Turner, 2008 [91]	Coronary heart disease, heart failure	The Netherlands	Economic evaluation	Nurses	Peripatetic nurse specialists	Adults with a diagnosis of coronary heart disease or heart failure	Not applicable – an economic evaluation of nurse-led assessment, investigation, and coordination of care for heart disease and heart failure
Kenealy, 2004 [92]	Diabetes	New Zealand	Longitudinal survey design	Not applicable – exploratory study	Not applicable	Not applicable – a survey exploring the barriers to diabetes management delivered by practice nurses	Not applicable – an exploration of practice nurse perspectives towards the delivery of diabetes care by practice nurses
Stenner, 2011 [93]	Diabetes	UK	Qualitative research	Not applicable – exploratory qualitative study	Not applicable	Patients with diabetes	Not applicable – an exploration of patient perspectives towards nurse prescriber-delivered care for diabetes
Wald, 2004 [94]	Heart failure	UK	Non-randomised experimental study	Nurse practitioner and GPs	Nurse Practitioner	Patients with heart failure	Primary care-led beta blocker clinic for patients with heart failure
Alves, 2021 [95]	Hypertension	Portugal	Non-randomised experimental study	Family practice nurses	Classification not specified	Patients with hypertension	Delivery of an educational tool for mental adjustment by nurses
Christianson, 1997 [96]	Hypertension	USA	Not defined	Practice nurses	Classification not specified	Patients with hypertension	Nurse-delivered hypertension self-management program
Persell, 2023 [97]	Hypertension	USA	Observational cohort study	Nurse care coordinators	Registered Nurses	Adults with hypertension that were eligible for Medicare	Remote patient monitoring of blood pressure and care coordination
Shaw, 2013 [98]	Hypertension	USA	Mixed methods	Administrators, physician/primary care providers (physician assistants), nurse practitioners, and IT professionals	Nurse Practitioner	Veterans with hypertension	Tailored telephone intervention with monthly calls: data entered by nurses and analysed by software that triggers modules of health education related to individual need
Stephen, 2018 [99]	Hypertension	Australia	Qualitative research	GPs and practice nurses	Classification not specified	Adults aged 45–74 with hypertension	Management of hypertension to improve medication management and modify lifestyle risk factors
Stephen, 2024 [100]	Hypertension	Australia	Qualitative research	Practice nurses	Classification not specified	Patients with hypertension at risk of cardiovascular disease	Nurse-led chronic disease management support (goal setting, lifestyle advice, development of an action plan)
Kyle, 2024 [101]	Insomnia	UK	Mixed-methods research	Practice nurses and research nurses	Registered Nurses	Adults with insomnia	Sleep restriction therapy
Sandlund, 2017 [40]	Insomnia	Sweden	Randomised controlled trial	Practices nurses and one physiotherapist	Classification not specified	Patients with insomnia	Nurse-delivered group CBTi for insomnia
Van der Zweerde, 2020 [102]	Insomnia	The Netherlands	Randomised controlled trial	Mental health nurse practitioner	Mental health trained nurses - classification not specified	Adults with insomnia	Cognitive behavioural therapy for insomnia
Wright, 2001 [103]	Ischaemic heart disease	UK	Qualitative research	Practice nurses	Classification not specified	Patients with established ischaemic heart disease	Assessment and management of patients with Ischaemic heart disease
Ciccone, 2010 [104]	Multiple conditions: cardiovascular disease, diabetes, heart failure	Italy	Non-randomised experimental study	Care managers (all nurses)	Research nurses, classification not specified	Individuals with cardiovascular disease, diabetes, or heart failure	Coordination of care by a nurse case manager
Katon, 2010 [105]	Multiple conditions: diabetes, coronary heart disease	USA	Randomised controlled trial	Primary care physicians and practice nurses	Registered Nurses	Patients with diabetes, coronary heart disease or both, coexisting with depression	Self-management program consisting of medication support and goal setting
Walker, 2014 [106]	Multiple conditions: type 2 diabetes, hypertension	New Zealand	Non-randomised experimental study	Nurse practitioners	Nurse Practitioner and practice nurses - classification not specified	Patients with uncontrolled type 2 diabetes and/or hypertension that have poor clinic attendance - makes them at risk of developing chronic kidney disease	Provision of education and development of care plans specific to individual patients
Kendall, 2010 [107]	Multiple conditions: type 2 diabetes, asthma, COPD, coronary heart disease	UK	Qualitative research – case study approach	Practice nurses, a Nurse Practitioner, and a health care assistant	Nurse Practitioner and practice nurses- classification not specified	Patients with type 2 diabetes, asthma, COPD, or coronary heart disease	Two separate chronic disease management clinics
Eley, 2013 [50]	Multiple conditions: type 2 diabetes, hypertension, ischaemic heart disease	Australia	Mixed methods	Practice nurses and GPs	Registered Nurse	Adults with at least one of the following conditions: type 2 diabetes, HT, or ischaemic heart disease	Practice nurse-led collaborative care for chronic disease - patient visits, education, GP referrals and case conferences with the GP
Hegney, 2013 [108]	Multiple conditions: type 2 diabetes, stable ischaemic heart disease or hypertension	Australia	Qualitative analysis of a randomised controlled trial	Practice nurses, GPs, and practice managers	Registered Nurse	Adults with at least 1 of the following conditions: type 2 diabetes, stable ischaemic heart disease, or hypertension	Practice nurse-led chronic disease management
Soejbjerg, 2024 [109]	Multiple conditions: type 2 diabetes, ischaemic heart disease	Denmark	Qualitative research to assess feasibility	Practice nurses and GPs	Classification not specified	Adults with type 2 diabetes and/or ischaemic heart disease who attended a yearly chronic disease assessment	Problem solving therapy to treat poor mental well being
Verwey, 2016 [110]	Multiple conditions: COPD, type 2 diabetes	The Netherlands	Process evaluation of a randomised controlled trial	Practice nurses	Classification not specified	Adults aged between 40–70 with COPD or type 2 diabetes	Practice nurse-delivered self-management support for increasing physical activity
Weise, 2023 [111]	Multiple conditions: type 2 diabetes, hypertension	Germany	Qualitative research	Not applicable – exploratory qualitative study	Advanced practice nurses or practice nurses – classification not specified	Patients with type 2 diabetes and/or hypertension	Not applicable - an exploration of patient acceptability of practice nurse-led dose adjustments for long term medications
Clarke, 2019 [112]	Prostate cancer	UK	Process evaluation	Practice nurses and GPs	Classification not specified	Men who have survived prostate cancer	Online health needs assessment for prostate cancer management and self-management
Clarke, 2020 [113]	Prostate cancer	UK	Non-randomised experimental study	Practice nurses	Classification not specified	Patients with a diagnosis of prostate cancer	Online prostate cancer holistic needs assessment supported and reviewed by practice nurses
Watson, 2018 [114]	Prostate cancer	UK	Mixed methods research	Practice nurses and primary care research nurses	Classification not specified	Prostate cancer survivors	Face to face and/or telephone follow up for prostate cancer support and education
Afzali, 2013 [115]	Type 2 diabetes	Australia	Economic evaluation	Practice nurses	Classification not specified	Patients with type 2 diabetes	Not applicable – an economic evaluation of nurse-delivered diabetes management
Avery, 2016 [116]	Type 2 diabetes	UK	Systematic development process	GPs, practice nurses, and health care assistant	Classification not specified	Adults with type 2 diabetes	Movement as Medicine for Type 2 diabetes intervention for behaviour change
Birgisdóttir, 2025 [117]	Type 2 diabetes	Iceland	Retrospective cohort study	Diabetes nurses	Classification not specified	Adults with type 2 diabetes	Not applicable – retrospective evaluation of the delivery of care for type 2 diabetes
Blackberry, 2013 [118]	Type 2 diabetes	Australia	Randomised controlled trial	Practice nurses	Classification not specified	Patients with type 2 diabetes	Telephone-delivered diabetes coaching
Boyle, 2016 [119]	Type 2 diabetes	Australia	Qualitative research	Not applicable – exploratory qualitative study	Not applicable	Patients with type 2 diabetes	Not applicable - an exploratory qualitative study of patient perspectives towards practice nurse-delivered diabetes management
Carlisle, 2013 [120]	Type 2 diabetes	Australia	Qualitative research	Diabetes care coordinator nurses	Classification not specified	Patients with type 2 diabetes	Telehealth visits for diabetes monitoring and support
Chmiel, 2017 [121]	Type 2 diabetes	Switzerland	Cross sectional study	Practice nurses	Registered Nurse, Enrolled Nurse and practice nurses - classification not specified	Adults with diabetes	Delivery of type 2 diabetes care using the chronic care model
Chimoriya, 2024 [122]	Type 2 diabetes	Australia	Qualitative research	Not applicable – exploratory qualitative study	Classification not specified	Adults with type 2 diabetes	Counterweight program for weight loss management
Crowley, 2013 [123]	Type 2 diabetes	USA	Randomised controlled trial	Nurses working with general practice but remotely	Research nurses, classification not specified	African American patients with type 2 diabetes	Nurse-delivered telephone self-management support - monthly self-management support and three-monthly medication management support
Dellasega, 2010 [124]	Type 2 diabetes	USA	Qualitative research	Registered nurses	Registered Nurses	Patients with type 2 diabetes	Nurse-delivered motivational interviewing to increase self-care for type 2 diabetes
Furler, 2014 [48]	Type 2 diabetes	Australia	Process evaluation of a feasibility study	GPs and practice nurses	Classification not specified	Individuals with type 2 diabetes that were at maximal oral therapy and required insulin therapy	New model of care for insulin commencement
Gianfrancesco, 2020 [125]	Type 2 diabetes	UK	Qualitative research	Not applicable – exploratory qualitative study	Not applicable	Patients with type 2 diabetes	Not applicable – an exploration of the delivery of diabetes nutrition education by practice nurses
Gilles de La Londe, 2023 [126]	Type 2 diabetes	France	Quasi-experimental study	GPs and advanced practice nurses	Advanced Practitioners Nurses	Patients with type 2 diabetes	New model of care for the management of type 2 diabetes, including physical assessment and patient education
Goff, 2020 [127]	Type 2 diabetes	UK	Qualitative research	Not applicable – exploratory qualitative study	Not applicable	African and Caribbean adults with type 2 diabetes	Not applicable – an exploration of the perspectives of practice nurses, GPs, diabetes nurses, and diabetes dieticians towards the challenges to the management of diabetes in general practice
Graves, 2016 [128]	Type 2 diabetes	UK	Qualitative research	Practice nurses	Classification not specified	Patients with sub-optimal management of type 2 diabetes	Motivational interviewing with patients - with monthly supervision by a psychologist
Greaves, 2003 [129]	Type 2 diabetes	UK	Qualitative research	Not applicable – exploratory qualitative study	Not applicable	Patients commencing insulin treatment	Not applicable – an exploration of the perspectives of practice nurses towards their involvement in insulin commencement for people with type 2 diabetes
Hardeman, 2014 [130]	Type 2 diabetes	UK	Qualitative research	Practice nurses	Classification not specified	Patients with type 2 diabetes	Nurse-led behaviour change intervention by practice nurses to improve medication adherence
Helmink, 2012 [131]	Type 2 diabetes	The Netherlands	Mixed methods research	GPs, physiotherapists, and practice nurses	Classification not specified	Patients with high fasting glucose levels and type 2 diabetes	Lifestyle behaviour change using coaching and motivational interviews
Hogervorst, 2021 [132]	Type 2 diabetes	The Netherlands	Qualitative research	Nurses, GPs, and pharmacists	Nurse Practitioner	Patients with type 2 diabetes that were non-adherent with medications and were poorly controlled	Nurse-practitioner support to better manage medications and diabetes self-management
Ismail, 2018 [133]	Type 2 diabetes	UK	Randomised controlled trial	Practice nurses	Classification not specified	Adults aged 18–79 with type 2 diabetes for at least 2 years and with suboptimal glycaemic control	Cognitive behavioural therapy, motivational interviewing
Jansink, 2010 [134]	Type 2 diabetes	The Netherlands	Qualitative research	Not applicable – exploratory qualitative study	Not applicable	N/A as exploration of current practice prior to implementation but intended to inform care for patients with type 2 diabetes	Not applicable – qualitative exploration of registered nurse’s perspectives towards delivering lifestyle counselling for activity
Jansink, 2013 [135]	Type 2 diabetes	The Netherlands	Randomised controlled trial	Nurses	Classification not specified	Patients with type 2 diabetes	Nurse-delivered motivational interviewing for lifestyle counselling
Kolltviet, 2024	Type 2 diabetes	Norway	Qualitative research	Practice nurses, diabetes nurse specialists, GPs and	Classification not specified	Patients with type 2 diabetes	Guided Self-Determination to support patients to self-manage their diabetes
Nissen, 2024 [136]	Type 2 diabetes	Denmark	Qualitative research	Not applicable – exploratory qualitative study	Not applicable	Patients with type 2 diabetes	Not applicable – qualitative exploration of nurse and GP perspectives towards the use of a diabetes tool (Diabetes Control Support Tool)
Odnoletkova, 2016 [137]	Type 2 diabetes	Belgium	Mixed methods	Nurses and GPs	Certified diabetes educators	Patients with type 2 diabetes	Telephone-delivered diabetes coaching
O’Hare, 2004 [138]	Type 2 diabetes	UK	Randomised controlled trial	Asian link workers, practices nurses, and a community diabetes specialist nurse	Practice nurses - classification not specified, and community diabetes specialist nurse	Individuals who had South Asian ethnicity, type 2 diabetes with either hypertension and/or hypercholesterolaemia	Diabetes management with additional nurse appointment weekly, support from an Asian link worker and access to support from a community diabetes nurse specialist
Oyegbami, 2019 [139]	Type 2 diabetes	USA	Mixed methods research	Nurse practitioners, a dietician, and a social worker	Nurse Practitioner	Hispanic adults with type 2 diabetes	Bi-weekly telephone-delivered support to improve glycaemic control
Poskiparta, 2006 [140]	Type 2 diabetes	Finland	Qualitative research	GPs and practice nurses – diabetes educator	Classification not specified	Patients with type 2 diabetes or impaired glucose tolerance	Dietary and physical activity counselling
Ratanawongsa, 2012 [141]	Type 2 diabetes	USA	Survey design after randomised controlled trial	Physicians, resident physicians, nurse practitioners, and physician assistants	Nurse Practitioner	English, Spanish, or Cantonese-speaking adults with type 2 diabetes with a recent haemoglobin A1c (HbA1c) *≥* 8.0%, and at least 1 primary care visit at one of the four participating clinics	Automated telephone-delivered diabetes self-management care with nurse support
Rehackova, 2022 [142]	Type 2 diabetes	UK	Mixed methods research	Practice nurses, dieticians, and GPs	Classification not specified	Adults aged 20–65 years with a BMI of 27–45 kg/m2 diagnosed with type 2 diabetes within the previous 6 years	Counterweight program for weight loss management
Rehackova, 2022a [143]	Type 2 diabetes	UK	Qualitative research	Practice nurses, dieticians, and GPs	Classification not specified	Adults aged 20–65 years with a BMI of 27–45 kg/m2 diagnosed with type 2 diabetes within the previous 6 years	Qualitative exploration of the experiences of patients who were delivered the Counterweight program for weight loss management
van Bruggen, 2008 [144]	Type 2 diabetes	The Netherlands	Randomised controlled trial	GPs, practice assistants, and nurses	Classification not specified	Patients with type 2 diabetes	Implementation of locally adapted diabetes guidelines
Weinberger, 1995 [145]	Type 2 diabetes	USA	Randomised controlled trial	Practice nurses	Classification not specified	Patients with type 2 diabetes	Telephone-delivered case management (2.5 h/year)
Wolf, 2014 [146]	Type 2 diabetes	USA	Quasi-experimental - no control	Nurses	Classification not specified	Patients with type 2 diabetes	Face-to-face or telephone-delivered diabetes action planning and behaviour change
Woodcock, 1999 [147]	Type 2 diabetes	UK	Randomised controlled trial	Nurses	Classification not specified	Patients with type 2 diabetes	Patient-centred care for diabetes management

*Listed by condition and in alphabetical order

**Table 3:** Data synthesis table

Barriers	Facilitators
**External context** ***Policy and legislation*** *(Lack of legal frameworks)* Lack of legal framework to support delivery of the intervention [136] Insufficient legislation to enable the development of sustainable services [120] ***Stakeholder buy-in*** *(Lack of funding by insurance companies)* Lack of financial agreements with insurance companies for the funding of the intervention [75] ***Fit with local or national agenda*** *(Timing of intervention)* Introduction of the model of care at a time of concern about the nursing profession [78] Implementing a model of care during restructuring of primary care services reduces engagement by practices due to uncertainty [132] *(Impact of COVID-19)* Infection risk limited capacity to provide hands-on care [117] Infection risk limited face-to face appointments [122] COVID-19 increased workloads of general practice staff [145] Lockdowns due to COVID-19 made delivery of an intervention challenging [101] ***Infrastructure*** *(Software infrastructure)* Lack of standardised databases and software in general practice [79] Lack of centralised IT infrastructure to support ongoing delivery of the intervention [98] Requiring institutional approval is a barrier to making change to electronic medical software [86] *(Changing context)* General practice is an environment of constant change [76, 126] ***Economic climate and governmental financing*** *(Established finding models that provide insufficient funding)* Limited funding models [79, 120] Insufficient funding for the intervention [73] *(Ineffective funding models)* Funding model incentivises short consultation times which limits the delivery of interventions that take time to deliver[73] Funding model does not suit multidisciplinary models of care[120, 136] *(Lack of funding for care delivery)* Lack of funding for nurse-delivered care [80, 89, 100, 108, 121] Lack of funding for the intervention [88] Lack of funding for team-based activities delivered by nurses [84] ***Financial incentives*** *(Inadequate financial incentive)* Lack of financial incentive for the practice to deliver the intervention [73, 142] Inadequate financial renumeration for nurses to reflect the increased responsibility associated with new models of care [80] *(Financial incentive that does address patient need)* Practice incentives that offer financial incentive but do not address the needs of patients [126]	***Policy and legislation*** *(Governmental support of the intervention)* Government recognition of the need for the intervention [120] *(Institutional support)* Less institutional support is associated with greater motivation by practices to implement and maintain an intervention [130] ***Stakeholder buy-in*** *(Support from administration)* Agreement by administration that intervention is valuable [98] Agreement by administration that the model of care fits within the values of the organisation [98] ***Infrastructure*** *(Software infrastructure)* Adequate IT infrastructure [120] *(Practice size)* Larger-sized practices are associated with greater confidence in nurses [83] Larger-sized practices enable greater support for nurses from colleagues [75] ***Economic climate and governmental financing*** *(Funding for models of care)* Consistent funding for practice nurse activities within general practice [79] Funding of interventions to fund the salaries of practice nurses [48] Adequate funding is a facilitator to delivering long appointments [145] Funding for nurse-delivered interventions [84, 100] Nurse-delivered care is cheaper than GP-delivered care [111] ***Financial incentives*** *(Renumeration of nurses)* Increased financial renumeration for nurses to reflect increased responsibility associated with new models of care [128] Standardised renumeration for practice nurses [79] Renumeration for nurses that includes support to undertake professional development [79]
**Organisation** ***Resources*** *(Time)* Limited time to deliver the intervention due to existing busy workloads [70, 71, 73, 74, 76, 80, 82–84, 88, 95, 97, 99–101, 124, 126–128, 131, 139, 142, 144, 145] Busy workloads make finding time to establish the model of care challenging [80, 91, 136] Lack of time for team meetings to discuss implementation [76] Lack of time to practice delivering the intervention prior to implementation [119] Limited time to provide care for patients with complex health needs [71] Lack of time in appointments means other urgent clinical issues take priority [76] A lack of clinical time reduces nurse confidence in the delivery of the intervention [83] Lack of time limits nurse capacity to seek out support from specialist staff [124] Busy workloads limit GP capacity to support nurses to deliver the intervention [128] Limited time capacity despite available funding [118, 132] Limited time to attend training [83, 91, 116, 126, 133] Busy workloads limit nurse capacity to provide patient centred care [133] Complex processes associated with models of care add demand to an already busy practice [69] Implementing new interventions increases workloads [49, 69, 76, 118] *(Insufficient staffing)* Nursing staff shortages [91] Staff turnover increases workloads for remaining staff [109] Lack of nurses trained to deliver the intervention [73] Difficulties finding staff to cover for nurses to attend training [71] Lack of staff over leave periods halts delivery of care [117] Insufficient administrative support [91] *(Financial cost to the practice)* Loss of income related to staff attending training [133] Cost of training staff [82, 112] Cost of the equipment needed to deliver the intervention [73] Cost of set up for the model of care without established funding [121] *(Lack of physical space)* Lack of appropriate private space to deliver the intervention [70, 72, 73, 80, 91, 118, 121] *(Information technology)* IT connectivity issues limit use of technology [110] Lack of capacity within electronic medical record systems to screen for suitable patients [131] Slow internet access [112] Computers ill-equipped for use in delivery of the intervention [82] *(Equipment)* Access to poor quality equipment only [83] Lack of access to the equipment needed to deliver the intervention [71, 73] ***Processes and systems*** *(Length of appointments)* Short appointments are a barrier to patient engagement [126] Short appointments with GPs are a barrier to communication by the patient [100]Short appointment times are insufficient for effective delivery of the intervention [69, 119] Short appointment times limit the development of trust between the health care professional and the patient [126] Long appointments are expensive to deliver [145] *(Appointment scheduling)* Inflexibility in nursing appointment schedules limits patient access to care [107] Lack of communication about appointment scheduling for GPs and nurses increases work pressure for GPs [145] Misalignment of GP and nurse schedules is a barrier to collaborative practice [145] Difficulty scheduling long appointments into existing short consultation times [101] *(Structure of chronic disease management in the practice)* Lack of a structured approach to chronic disease management with adequate protocols [75] Nurses working for practices without a structured approach to chronic disease management experience a lack of confidence in care delivery [75] ***Culture*** *(Priorities of the practice)* Changing priorities within the practice[91] ***Relationship*** *(Communication with external stakeholders)* Lack of established relationships with key local stakeholders [131] *(Relationships with specialist providers)* Poor relationship between the general practice team and specialist teams [86] Poor communication between general practice and specialist services is a barrier to accessing support from specialist services [112] Limited communication from hospitals [86] Minimal communication with specialist care results in a lack of clarity about the role of the specialist provider in care delivery [124] A limited relationship with specialist providers limits nurse capacity to determine their competence in the delivery of the intervention [124] *(Health care professional-patient relationship)* Delivering a new intervention to patients that nurses have known for a long time can be difficult [110] An ineffective nurse-patient relationship is a barrier to patient engagement [132] Concern that delivery of the intervention could damage the health care professional-patient relationship [112, 133] ***Skill mix*** *(Role clarity)* Lack of clarity about team members roles [76, 133] A lack of definition of team members’ roles can contribute to poor collaboration between nurses and GPs [80, 145] *(Introduction of the role)* Lack of consultation about the scope of the new nurse role can lead to concern about its impact on the role of other team members [78] Lack of a formal introduction of the nursing role results in a lack of clarity about how the role fits into the broader team [78] *(Delegation of responsibility)* Delegation of responsibilities to the nurse can result in a loss of GP knowledge [71, 76] *(Staffing)* Part time staffing limits capacity to provide prompt patient follow up [107, 128] Insufficient trained staff results in cessation of the intervention in the case of staffing changes [91] Staffing changes during the delivery of the intervention [77, 86, 121] Delivery of the intervention by nurses employed outside of the general practice limits the development of the nurse-patient relationship [113] Delivery of care by nurses working remotely and not employed by the practice limits relationships with GPs [146] Insufficient opportunities to deliver the intervention can result in difficulty maintaining competency [114, 128] *(Inconsistent delivery of care)* Inconsistent approach to delivery of the intervention by team members [76] ***Involvement*** *(Teamwork)* Lack of teamwork [73, 79, 139] Lack of involvement of administrative staff in the model of care [70] Lack of support from the practice manager to implement the model of care [100] Lack of support from team members is associated with time pressure in nursing appointments [124] Working alone on the model of care limits a nurse’s capacity to receive clinical support from colleagues [83] Working alone on the model of care limits a nurse’s ability to ascertain their competence in the delivery of the intervention [91, 128] Working collaboratively with nurses increases GP workloads [84] *(GP support of the model of care)* Lack of GP support for the implementation of the model of care [100] Lack of GP cooperation in the model of care increases nurse time in the delivery of the intervention [136] Lack of GP support for implementation of the model of care limits the capacity of nurses to be involved [82, 131] Lack of support from the GP reduces nurse interest in being involved in the model of care [127] Lack of support from the GP is a barrier to nurse confidence in the delivery of the intervention [83, 124] Lack of support from the GP leads to feelings of isolation in nurses [91] Lack of allocated time with the GP to discuss patient progress [83] *(Nursing peer support)* Lack of support from nursing colleagues is a barrier to nurse confidence in the delivery of the intervention [83] Lack of collegial support is a barrier to implementation by nurses [75] *(Hierarchical model)* Lack of autonomy by nurses to be flexible with appointment length [83] *(Stakeholder involvement in implementation)* Not having clinicians in planning meetings for the implementation of the intervention delays important changes to protocols [96]	***Resources*** *(Time)* Protected time to deliver the intervention [82, 128, 139, 147] Sufficient time for interpretation of results improves nurse confidence in the delivery of the intervention [83] *(Staffing)* Adequate staffing [73, 75, 99] Extra administrative assistance [70, 82, 127] A dedicated staff member to coordinate intervention delivery [97] *(Physical space)* Access to appropriate private space to deliver the intervention [82, 108] *(Financial cost)* Financial support for patients to access transport for appointments [137] *(Resources)* Access to existing resources for use in the intervention [98, 120] Sufficient equipment required to deliver the intervention [75] *(Technical support)* Ongoing IT support [112, 120] ***Processes and systems*** *(Length of appointments)* Longer appointment times facilitate the development of the nurse-patient relationship [103, 123] Longer appointment times facilitate patient engagement with models of care [50, 84, 94, 99, 100] Longer appointment times allow for more comprehensive delivery of the intervention [127] Longer appointment times enable the delivery of patient-centred care [108, 127, 145] *(Continuity of care)* Continuity of care is a facilitator for nurse engagement [91, 128] Continuity of care is a facilitator to success of the model of care [48] Continuity of care is a facilitator to patient engagement [108] Continuity of care is a facilitator to rapport with patients [140] Continuity of care is a facilitator to patient-centred care [140] Continuity of care facilitates trust in the nurse-patient relationship [94, 140] Continuity of care is not required for patient engagement if the patient perceives the intervention as task-orientated [119] Continuity in the working relationship between nurses and GPs facilitates teamwork [48] *(Compatibility with existing work processes)* Compatibility of the intervention with existing work practices [89, 130] *(Appointment scheduling)* Booking appointments ahead of time to improve patient attendance [137] Alignment of nurse availability with patient availability [49] Availability of appointments with nurses [97, 100] Flexibility in appointment scheduling [101] Conducting phone reminders to improve patient attendance at appointments [137] Sending prompts to patients [87] Scheduling of patient appointments by nurses could enhance the nurse-patient relationship [96] Regular appointments facilitate patient engagement [140] Regular appointments provide accountability for patients to adhere to treatment [122] *(Billing)* Consistent, efficient and transparent billing systems reduces clinician workloads [96] *(Organisation of the practice)* An organised practice is a facilitator to patient engagement [111] ***Culture*** *(Positive culture)* Positive workplace culture [76] Regular team meetings [96, 105] ***Relationship*** *(Relationship between practice team members)* Respect and trust between team members [76, 108, 111] *(Relationship between the nurse and GP)* Collaborative nurse-doctor relationship [104] Trust between nurse and GP facilitates teamwork [48] GP confidence in the nurse facilitates patient confidence in the nurse [108] Effective communication between GPs and nurses [100] Effective communication between GPs and nurses is a facilitator to a collaborative relationship [145] Face-to-face communication between GPs and nurses is a facilitator to a collaborative working relationship [145] *(Relationships with specialist providers)* An educational and clinical link between primary care and secondary care is a facilitator to the establishment of models of care [95] Involvement of a specialist nurse can facilitate patient engagement [147] A working relationship between a practice nurse and a specialist nurse facilitates education of the practice to deliver evidence-based care [106] Support from specialist staff improves practice staff confidence [112, 124, 127, 128, 141] Support from specialist staff improves practice workload [141] Involvement of a cultural link worker can facilitate patient adherence in culture-specific patient groups [147] *(Relationships with external stakeholders)* Prior experience with research is a facilitator to practice engagement in new models of care [98] A prior trusted relationship with the external provider introducing the model of care is a facilitator to practice engagement [120, 141] *(Health care professional-patient relationship)* Trust is a facilitator to patient engagement [48, 111, 140] Shared decision-making facilitates patient engagement with the intervention [74] A strong nurse-patient relationship is a facilitator to effective delivery of the model of care [104] A successful nurse-patient relationship improves patient engagement with the model of care [48, 109] Support and encouragement by health care professionals is a facilitator to patient engagement [116, 122, 140] Familiarity with the nurse is a facilitator to patient engagement [48] A pre-existing relationship with the patient is a facilitator to delivery of a model of care [109] A pre-existing relationship with the patient is a facilitator to determining suitability of an intervention [109] Effective communication by the nurse is a facilitator to patient confidence in the competency of the nurse [108] Accountability to the health care professional facilitates patient engagement [122, 140] Regular contact with the GP is a facilitator to patient engagement [111] Ongoing support from health professionals facilitates ongoing engagement by patients [122] Ongoing support from health professionals facilitates adherence to treatment [101] ***Skill mix*** *(Role clarity)* Defined roles for all team members [75, 79, 122] Defining the responsibilities of team members is a facilitator to collaborative care [145] Agreement about the accountability of the intervention [48] *(Delegation of responsibility)* Shared responsibilities between team members [50, 76, 108] Delegation of all responsibilities to the nurse facilitates a consistent approach to delivery of the intervention [76] *(Staffing)* Employment of staff who display teamwork skills [108] Nurses with less than eleven years of experience have greater confidence in delivery of an intervention [83] The delivery of a culturally-specific intervention by nurses with that cultural identity may facilitate the development of the nurse-patient relationship [126] The delivery of an intervention by a trained specialist nurse enables early initiation of treatment and patient support [85] ***Involvement*** *(Teamwork)* Effective teamwork within the practice [84, 103, 127, 128] Evidence of teamwork between nurses and GPs instils confidence in patients [50, 94, 99] Teamwork between nurses and GPs reduces duplication of care delivery [82] Working collaboratively increases the skills and knowledge of GPs and nurses [145] Collaboration between GPs and nurses improves the quality of the care that they deliver [125, 145] Support from colleagues increases staff motivation to deliver the intervention [130] *(GP support of the model of care)* Support from GPs improves nurse confidence in the delivery of the intervention [80, 83, 124, 128] GP support of the model of care facilitates delivery of the intervention by the nurse [91] GP support of the model of care increases patients’ confidence in the intervention [50, 88, 122] GP involvement in the model of care increases patients’ confidence in the intervention [97, 99, 100, 108] GP involvement in the model of care facilitates a collaborative approach to care delivery [82] A willingness by GPs to delegate chronic disease management to nurses is needed to sustain a model of care ongoing [91] Supervision of the nurse by the GP is a facilitator to patient engagement [111] *(Hierarchical model)* GP support of the model of care enables nurses to dedicate more time to the delivery of the intervention [131] Capacity for nurses to amend appointment length as needed [83] *(Nursing peer support)* Nursing team support improves nurse confidence [83, 101] *(Stakeholder involvement in implementation)* Engagement of individuals with decision making power in implementation meetings [96] *(Shared vision)* Agreement within the practice that the intervention should be delivered by nurses [116]
**Professional** ***Professional role*** *(Scope of practice)* Concerns by nurses about impact on professional indemnity insurance if their scope of practice is not clearly defined [128] Concern by nurses about working outside of their scope of practice [127, 132] Lack of confidence in nurse skills by GPs is a barrier to a greater role for nurses [73] Lack of definition of the scope of practice of the enhanced role of the nurse [70, 78, 96] Lack of clarity about the scope of practice of nurses is a barrier to patient engagement [111] Concern by patients that nurses could work beyond their scope of practice [111] Uncertainty about the appropriateness of nurse-delivered care is a barrier to patient engagement [111] *(GP attitudes towards delegation of responsibility to the nurse)* GP reluctance to delegate responsibility to the nurse due to lack of knowledge about the nursing scope of practice [80] GP reluctance to delegate responsibility to the nurse due to desire to provide chronic disease management themselves [91] GP reluctance to delegate responsibility to the nurse due to a fear of loss of control [121] GP reluctance to delegate responsibility to the nurse due to insufficient workload of their own [121] GP reluctance to delegate responsibility to the nurse due to lack of knowledge about the model of care [82] Insufficient nurse knowledge and skills reduces GP confidence in delegating responsibilities to nurses [76] *(Nurse-GP relationship)* Perception by nurses that GPs do not value their role within the model of care [73, 80, 127] A shift from a hierarchical relationship to a model with a nurse at the centre of care can be challenging for GPs [121] *(Perception of the nursing role by nurses)* Uncertainly by nurses about providing recommendations for care to GPs [136] ***Attitudes to change*** *(Staff disinterest in the model of care)* Lack of engagement by nurses and GPs [138] Lack of motivation by practice staff [142] Lack of interest in new models of care by GPs [91] A lack of interest by GPs in being involved in new models of care due to impact on existing work practices [80] Lack of interest by nurses in extending their scope of practice to deliver new models of care [80, 103] Uncertainty of the model of care by peers limits engagement by staff [141] Lack of mention of the intervention by GPs within consultations [102] *(Staff uncertainty about the effectiveness of the model of care)* Health care professional lack of confidence about the effectiveness of the intervention [96, 141] A perception by health care professionals that the intervention only benefits a subgroup of patients [87, 116] Perception by GPs that the intervention lacks benefit [96] Health care professional uncertainty about the benefit of the intervention is a barrier to delivery of the intervention by GPs and nurses [75] Clinician uncertainty of usability of patient resources [144] *(Perception of workload demands due to the model of care)* The demand of the model of care on the role of the nurse limits engagement by practices [77] Resentment about the additional workload associated with the model of care [132] Reluctance by nurses to engage with the model of care due to uncertainty about the time commitment needed [98] Uncertainty about how to cover staff leave [98] *(Uncertainty about the appropriateness of the model of care)* Health care professional lack of confidence about the appropriateness of the intervention [76, 142] Health care professional uncertainty about patient capacity to engage with an online intervention [112] Concern about the safety of the intervention is a barrier to staff engagement[141] Uncertainty about the appropriateness of the intervention for the patient group [110] *(Uncertainty about the value of the model of care)* Time invested in non-adherent patients is not seen as valuable for the clinical outcomes achieved [131] Uncertainty about the cost-effectiveness of the model of care [75, 131] Time invested in the implementation for minimal patients reduces GP engagement with the intervention [131] Telephone delivered interventions are not given the same level of priority as face-to-face interventions [88] *(Competing priorities)* Competing demands of general practice [71, 88, 98, 118, 136, 139] Fear by nurses that delivering the intervention could be perceived as if they are not prioritising their regular work [127] ***Philosophy of care*** *(Differing patient-clinician values)* Lack of engagement by patients due to a perceived lack of priority is a disincentive for nurse engagement [114] Lack of accountability for self-management by patients makes delivery of the intervention difficult[76, 126] A conflict between patient’s belief system and the intervention limits patient’s engagement with the model of care [126] A conflict between the patient’s cultural identity and the intervention [73, 126] Differing priorities for care between the patient and clinician [109] Differing expectations of the role of the GP between the GP and patient [109] Patient reluctance to change habits despite education from the nurse [103] *(Patient preference)* Patient preference not to participate in group treatment [116] Patient preference to only see GP for care [121] *(Approaches to care)* Lack of person-centred care reduces patient satisfaction [90] Lack of patient-centred care is barrier to patient engagement [109] ***Competency*** *(Insufficient training)* Insufficient training leads to lack of confidence in the delivery of the intervention [75, 83, 84, 103, 110, 132] Insufficient training limits capacity to deliver the intervention [73, 91, 97] *(Characteristics of training)* Time consuming training [84] *(Insufficient knowledge/skills)* Insufficient knowledge leads to a lack of confidence in the delivery of the intervention by nurses [124] Lack of knowledge about best practice care [86] Lack of knowledge limits the ability of GPs to support the nurse to deliver the intervention [83, 128] Lack of knowledge about relevant guidelines [86, 142] Lack of knowledge about the influences of culture on a patient’s approach to chronic disease management [139] Lack of knowledge about external organisations is a barrier to development of effective relationships with those organisations [78] Limited health professional communication skills [138] Lack of empathy due to lack of personal experience [133] Lack of GP knowledge about the referral process [97] *(Impact of lack of knowledge on delivery of the intervention)* Lack of knowledge limits capacity to deliver the intervention [112, 133, 139] Lack of GP knowledge about an intervention is a barrier to GP delivery of an intervention [135] Lack of knowledge about the chronic condition reduces the quality of care provided [103] Lack of knowledge about how to address non-adherence to the intervention [131] Lack of confidence in the competence of the nurse is a barrier to patient engagement [111] Evidence of lack of competence in health professionals is a barrier to patient engagement with the model of care [75] *(Impact of insufficient knowledge on workloads)* Updating knowledge to deliver interventions increases workloads [76] Lack of skills in nurses is associated with time pressure in appointments [124]	***Professional role*** *(Scope of practice)* Nurses working within their scope of practice facilitates GP support for nurse-delivered models of care [108] GP awareness of the scope of practice specific to nurse classifications [79] Presence of competency statements that define the scope of practice of the nurse [79] *(GP attitudes towards delegation of responsibility to the nurse)* Sufficient nurse knowledge and skills is a facilitator to delegation of responsibilities by GPs [76] *(Nurse attitudes towards their role)* Confidence in nurses to seek support from GPs [103] A commitment to their role is a facilitator to nurse engagement [80] ***Attitudes to change*** *(Staff interest in the model of care)* An interest in the intervention [73, 108] Females are more motivated to implement a model of care than men [130] *(Confidence in the model of care)* Belief that the implementation of the model of care will be successful [98] Belief by health care professionals that the intervention will improve patient outcomes [116, 145] Confidence in the model of care improves health care professional engagement [76] Belief in the value of the model of care [82] *(Perceived benefit to the model of care to scope of practice)* Models of care that enable nurses to have a broader scope of practice facilitate greater nurse engagement [98] Models of care that enable nurses to have a broader scope of practice facilitate greater job satisfaction for nurses [98] Having an opportunity to develop new skills is a motivator for nurses [91] *(Perceived benefit to the model of care to patients)* Receipt of patient feedback is a motivator for nurse engagement [80] Receipt of positive patient feedback is facilitator to nurse confidence in the delivery of the intervention [124] *(Appreciation for a need for the model of care)* Clinician awareness for a need for an intervention [100] *(Prior experience)* Previous experience in similar models of care enabled confidence in implementation [98] Experience delivering similar models of care that are considered valuable by practice staff [98] Greater self-efficacy prior to implementation is related to motivation to continue to deliver the intervention [130] *(Prior knowledge)* Knowledge about the intervention prior to implementation[89] ***Philosophy of care*** *(Alignment of patient-clinician values)* Perception of teamwork with the nurse improves engagement with the model of care by patients [108] Patient perception of nurse competence is a facilitator to a trusting nurse-patient relationship [131] Active engagement in the model of care by the nurse is a facilitator to a trusting nurse-patient relationship [131] Active engagement in the model of care by the nurse is a facilitator to patient engagement [84, 122] Motivation to change is a facilitator to engagement of nurses in the model of care [82] *(Approaches to care)* A person-centred approach to care is facilitator to patient engagement [69, 71, 84, 94, 99, 100, 108, 119, 123, 140] A person-centred approach to care facilitates the development of the nurse-patient relationship [69] An empathetic approach to care facilitates patient engagement with a model of care [104, 123] An empathetic approach by nurses enhances the nurse-patient relationship [103] A calm approach to care delivery facilitates ongoing patient engagement [123] An approachable communication style, without the use of technical language, enhances the nurse-patient relationship [99] A holistic approach to care is a facilitator to patient engagement [94] Shared decision making with the patient is a facilitator to patient engagement [84]A nursing approach to communication is a facilitator to patient satisfaction [111] Delivery of care by nurses is a facilitator to patient engagement with treatment [111] ***Competency*** *(Sufficient training)* Completion of training enables skill acquisition [116] Sufficient training enables confidence in the delivery of the intervention [80–82, 89, 101, 112] Sufficient training enables nurses to be confident in accepting accountability for care [128] Sufficient training is a facilitator to patient confidence in the nurse [108] Sufficient training can be a facilitator to patient engagement [147] Sufficient training increases awareness of the value of the model of care [116] *(Characteristics of training)* Ongoing training facilitates confidence in the delivery of interventions [83, 103, 128] Ongoing training support facilitates continued engagement by nurses [144] Education of the practice team facilitates a team approach to implementation [71, 82, 121] Formal training with supplemental practical supervised training facilitates competence [128] Formal training with supplemental practical on the job training improves engagement by general practice staff [141, 142] Practice delivering the intervention facilitates confidence in intervention delivery [116] The receipt of external training is a facilitator to nurse confidence in the delivery of the intervention [83] Formal cultural skills training is needed to successfully deliver culture-specific care [126] Providing professional development credit is a facilitator to clinician engagement in training [86] *(Sufficient knowledge and skills)* GPs with sufficient knowledge about the model of care can facilitate patient engagement [122] Confidence in a nurse’s competence is a facilitator to GP engagement [145] Nurses with adequate knowledge and skills require less guidance from GPs [135] *(Health professional skills)* Effective nurse communication skills [76] Capability to recognise psychological ill health in patients [148] Opportunities to exchange skills and knowledge between team members is a facilitator to staff engagement [145] Evidence of a nurse’s qualification is a facilitator to patient engagement [111]
**Intervention** ***Nature and characteristics*** *(Complexity of the intervention)* The intervention is complicated to deliver [73, 121] The intervention is too demanding for patients [97, 101] *(Time required to deliver the intervention)* The intervention is time consuming [83, 97, 136] A time consuming intervention is a barrier to patient engagement [75] An intervention that is delivered over a long time is a barrier to patient engagement [140] *(Customisation of the intervention)* Patient resources that cannot be adapted to meet individual patient needs have reduced use by patients [71, 87] Inadequate patient resources for patients with limited literacy [139] Inadequate patient resources for patients with a different first language [71, 139] *(Intervention design)* Scripted interventions are a barrier to person-centred care [129] Manual screening for suitable patients is laborious [131] Use of unfamiliar terminology is a barrier for some patients [69, 110, 126] Insufficient patient assessment within the model of care to adapt the intervention to patient need [110] Lack of immediate results limits patient engagement [133] *(Appointment scheduling)* Evaluating patient information and developing patient-specific plans is difficult to achieve in one appointment [69] Completion of detailed documentation during appointments is a barrier to delivery of the intervention [82] Infrequent appointments limit monitoring of patient progress [139] Appointment schedule does not align with existing work practices [89] *(Information technology)* Ineffective electronic medical software [86] Lack of access to intervention software by all staff [98] Intervention software that is not user-friendly [113] Incompatibility of intervention software with existing IT systems [98] Lack of integration into practice electronic medical record software can result in reduced use by clinicians [75, 113, 121] Poor integration of documentation with practice electronic medical software makes the delivery of the intervention more time consuming [71] Alert fatigue from software reminders [71, 98] *(Lack of benefit of the model of care)* Lack of financial benefit for GPs [91] Lack of evidence for the intervention is a barrier to patient engagement[75] *(Means of intervention delivery)* Telephone-delivered interventions are not a substitute for face-to-face delivered care [136] Telephone-delivered interventions limit the development of a trusting nurse-patient relationship [136] Telephone-delivered interventions reduce patient engagement with the intervention [122, 136] Patient engagement with online interventions can be poor due to being considered impersonal [75] *(Documentation)* Paper documentation is a barrier to integration of documents into practice electronic medical record software [136] Duplication of documentation [71] Documentation that does not align with clinical care [71] Lack of structured documentation [86] *(Intervention guidelines)* Inadequate guidelines for effective delivery of the intervention [133] *(Incompatibility with usual care)* Irrelevance of the model of care to the clinical need in appointments [76] *(Costs associated with the intervention)* Costs associated with an intervention are a barrier to patient engagement [49] Uncertainty about the cost of the intervention for patients limits staff engagement [97] *(Sustainability of the intervention)* Lack of sustainability is a barrier to patient engagement [140] ***Implementability*** *(Time requirements)* Extra time needed to embed the model of care into routine practice [50, 135] *(Workload demands)* Implementing a new model of care is time consuming [48, 70, 110, 136] Implementing a new model of care adds work to existing workload [99, 127] *(Timing of implementation)* A delay between training and implementation [101] Delays in implementation can result in loss of time allocated to the intervention [98] Delays in implementation can result in a loss in enthusiasm to implement the model of care [98] Delays in implementation following training can result in a loss of knowledge about the intervention [98] Commencement of implementation during holiday periods [86] *(Funding requirements)* Lack of funding to commence model of care [96] *(Training for the intervention)* Intensive training can be overwhelming for practice staff [141] Offering limited education within work hours [83] Offering limited dates for training can result in not all staff being able to be trained to implement the model of care [113] ***Safety and data privacy*** *(Security of intervention software)* Lack of security is a barrier to use of intervention software by patients [75]	***Nature and characteristics*** *(Intervention design)* Models of care that facilitate the delivery of patient-centred care by nurses [89] A model of care that facilitates an empathetic approach to care facilitates health care professional engagement with a model of care [76] A model of care that enables sharing skills and knowledge between health care professionals is a facilitator to staff engagement [145] Opportunity for patients to connect with other patients receiving the intervention [48, 75] [140] Alignment of the model of care with the underlying philosophy of nursing care [89] A model of care that facilitates shared care with specialist services and the patient is a facilitator for nurse engagement [112] Delivery of the intervention with other established interventions that are seen as valuable [98] A model of care that does not require a significant investment of time is a facilitator for GP engagement [136] Access to data related to patient progress provides motivation to nurses [87, 136] Structured follow up care facilitates patient motivation [99] Models of care that produce quick results are motivating for patients [122] Models of care that can be adapted to meet individual patient needs facilitate patient engagement [101] *(Appointment scheduling)* Introduction of the intervention over several appointments reduces the risk of overwhelming patients [48] Frequent appointments support the delivery of self-management care to patients [88] Regular appointments enable greater patient accountability for their role within the model of care [108] Scheduling appointments on specific times and days improves practice organisation [101] *(Information technology)* Linkage with practice electronic medical records software [71, 73, 87, 89, 112] Remote monitoring of patient progress facilitates nurse delivery of the intervention [110] Capacity to generate succinct reports from software [87] Capability to complete documentation online prior to appointments is a facilitator for patient engagement [112] A shared medical record facilitates collaboration between nurses and GPs [145] *(Evidence of benefit)* Evidence of benefits to patients [89, 91, 104, 120, 122] Models of care with tangible results facilitate patient engagement [122] Perceived benefits of the model of care for patients facilitates engagement of health care professionals [91, 96, 98, 112, 120, 124] A demonstrated evidence base for the model of care improves practice engagement with implementation [141] Perceived benefits of the model of care to general practice staff [130] A model of care that reduces GP workload is a facilitator to staff engagement [101, 108] Models of care that add value to the role of the nurse [89] Demonstration of evidence about the intervention facilitates patient engagement [140] Evidence of benefit provides motivation to patients [140] *(Means of intervention delivery)* Telephone-delivered interventions work for case management delivered to large geographical populations [143] Telephone-delivered interventions work for busy patients [72] Telephone-delivered interventions work for stable patients [72] Telephone-delivered interventions are convenient for patients [98, 113, 136] Telephone-delivered interventions are cost effective [98] Online-delivered interventions are time efficient [102] Face-to-face delivery of an intervention is suitable for patients with unstable conditions [72] Face-to-face delivery of an intervention facilitates the nurse-patient relationship [72, 112] Face-to-face delivery of an intervention facilitates motivation in patients [101, 122] *(Intervention-specific resources)* Structured protocols [79, 89, 99, 108, 109, 119, 131, 134, 135, 147] Use of structured protocols is a facilitator to patient confidence in nurse delivery of care [111, 135] Use of structured protocols is a facilitator to nurse confidence in the delivery of an intervention [135] Use of structured protocols can reduce the frequency of appointments [135] Use of structured protocols is a facilitator to nurse autonomy [135] Use of structured protocols is a facilitator to increased knowledge and skills of nurses [84] Easy to use resources [71, 75, 89, 113] The provision of guidelines for management of non-adherence to the intervention [131] Patient resources that can be adapted to meet individual patient needs [40, 48, 75, 126] Nurse involvement in the development of resources enables the development of patient-centred resources [40] Provision of model of care-branded stationary to patients is a motivator for patient engagement [87] Use of a standardised approach to documentation facilitates delivery of care by GPs and nurses [145] Development of protocols by GPs is a facilitator to patient engagement [111] *(Compatibility with usual care)* Alignment of the model of care with existing work practices [69, 71, 75, 98, 116, 120] *(Costs associated with the intervention)* A no cost intervention is preferred by patients [75] The provision of resources to patients at no cost is a facilitator for GP engagement [96] Subsidised treatment is a facilitator to patient engagement [122] ***Implementability*** *(Time requirements)* Minimal time needed for implementation can be a facilitator to maintaining enthusiasm in implementation [98] An initial investment of time allows for the nurse to develop confidence in the delivery of the intervention [108] *(Financial viability)* Practice nurse delivered care is economically viable [115] The delivery of an intervention by a trained specialist nurse is economically viable [92] *(Intervention-specific training)* Training that is offered with multiple modes of delivery [79] Training that is available locally [73] Training to use the technology associated with the intervention [120] The provision of initial training and guidance enables the implementation of models of care in practices without existing infrastructure [141]
**Patient** ***Capability to participate*** *(Social determinants)* Language barrier [75, 89, 112, 131, 133, 139] Low literacy [133, 149] Illiteracy [75] Limited health literacy [89, 139] Presence of stress [133] Psychological ill health [148] Low socio-economic status [89, 133] Low intelligence [127] Competing caring responsibilities [48, 126] Lack of access to the resources needed to participate in the intervention [89, 133, 137] Lack of access to the technology needed for the intervention [87, 112, 135] Lack of time to commit to the intervention [87, 122, 137] Lack of access to transport [117] *(Health related determinants)* Competing health priorities [48, 69, 82, 100, 101, 118, 119] Multiple health issues [89, 131] Receipt of interventions for other health conditions with advice that conflicts with the model of care [90, 107] Physical ill health [116, 148] Visual impairment [97, 112] Pain that impairs capacity to engage with the intervention [112] Addiction [131, 133] Advanced age [75] *(Patient skills)* Technology illiteracy [112, 135] Limited conversational skills [138] *(Psychological attributes)* Lack of confidence in their role in the intervention [76] Lack of discipline [133] Lack of self-efficacy [101] ***Relevance to self*** *(Patient attitudes towards the model of care)* Uncertainty about the benefits of the model of care [111] *(Belief about the relevance of the intervention to their condition)* A belief that their condition is untreatable [103] A belief that their condition is too longstanding to be suitable for the intervention [112] A belief that their condition is low priority [73] A belief their condition is mild and doesn’t require treatment [69, 75, 112] A belief that their condition is not serious enough to need the intervention [71] *(Belief about the relevance of the intervention to their circumstances)* The intervention is not a priority in the context of other commitments [127] A belief that the intervention does not meet their needs [87] A belief that they are too old for the intervention [69] A belief that they do not need the intervention [87] ***Willingness to participate*** *(Engagement with treatment)* Non-attendance at appointments [75, 76, 127, 136, 139] Non-adherence to treatment [75, 93] Lack of engagement with the model of care [71, 93, 97, 132] Lack of engagement by patients leads to lack of motivation by staff [133] Unwillingness to engage with the model of care [121] Not prioritising the intervention [101] *(Readiness to make change)* Lack of motivation [76, 136] Unreadiness to make change [82, 123, 127] *Confidence in the intervention)* Lack of confidence in the feasibility of the intervention [122] *(Fear and social pressures)* Fear of social stigma [73, 126] Fear of the intervention [76, 126] Avoidance of the intervention due to it being an unwelcome reminder of their health issues [112] Peer pressure by others not to participate [133] *(Altruism)* Awareness of the demands on GPs limits patient engagement due to concern about burdening the GP [109] Table 3: Legend **Primary themes** ***Secondary themes*** *(Derived sub-themes)*	***Capability to participate*** *(Social determinants)* Support from family facilitates adherence to treatment [122, 140] *(Health-related determinants)* Patients that are managing well with their condition [103] Patients that perceive themselves as having a stable condition [108] *(Patient skills)* Technology literacy [120] *(Psychological attributes)* Self-motivation [101] An intrinsic desire to engage with a model of care [140] ***Relevance to self*** *(Patient attitudes towards the model of care)* A belief by patients that the intervention is useful [112] A belief by patients that the intervention is needed [97] A belief that they would receive faster treatment [111] ***Willingness to participate*** *(Altruism)* A belief that the model of care frees up health professional time [50, 108, 112] To support a greater role for general practice nurses [111] *(Readiness to make change)* A desire to make change [100] *(Motivations to participate)* To improve their health status [100, 122, 140] To improve the symptoms of their chronic condition [140] To reduce their use of medication [122] To improve their knowledge about their chronic condition [100]

## Results

### Study selection

**Fig. 1:**
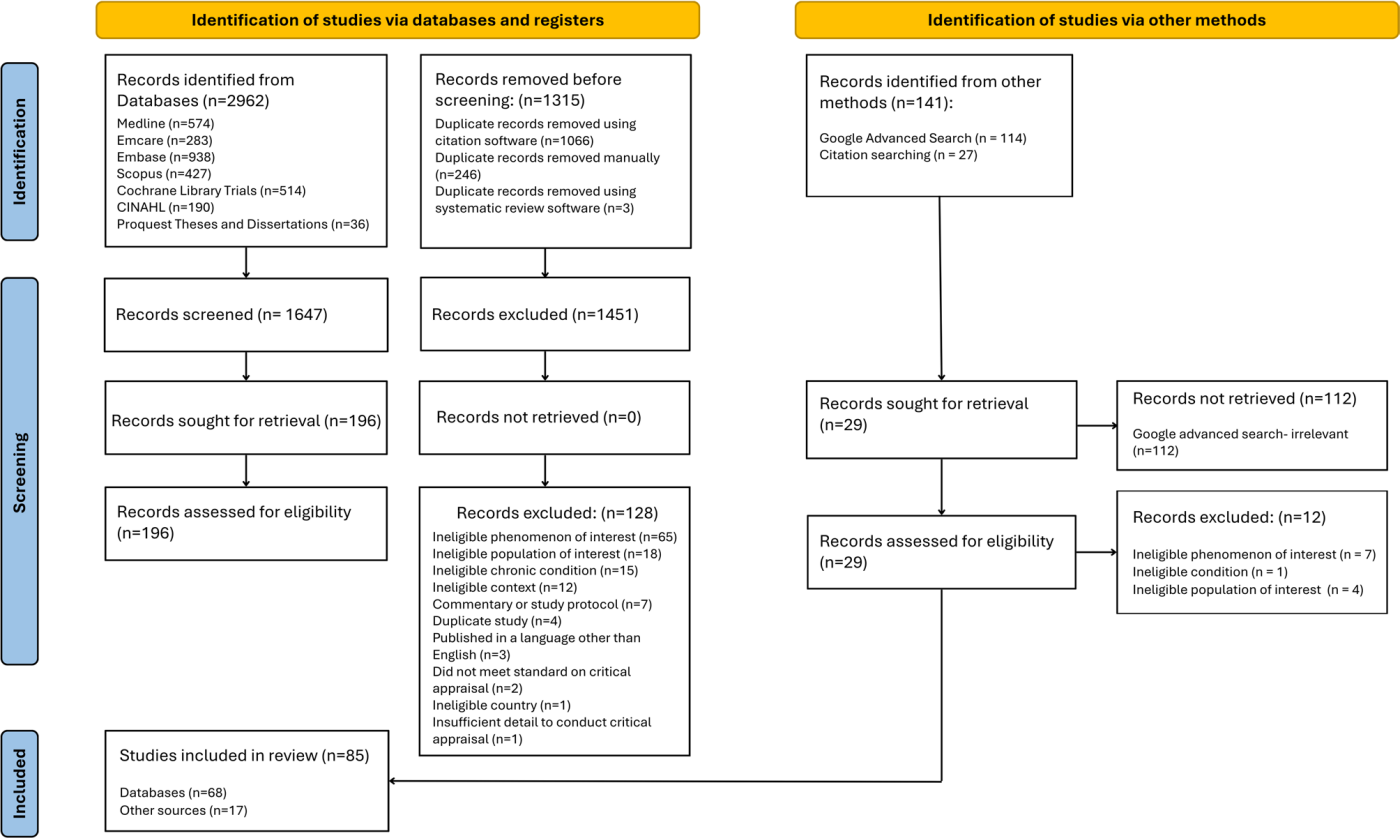
PRISMA flow diagram

Eighty-five records were included in the review, of which 68 were identified from database searches and 17 were identified from other sources. Greater detail about the search and screening process is presented in the PRISMA flow diagram (Fig. 1) [51].

### Characteristics of included records

**Fig. 2:**
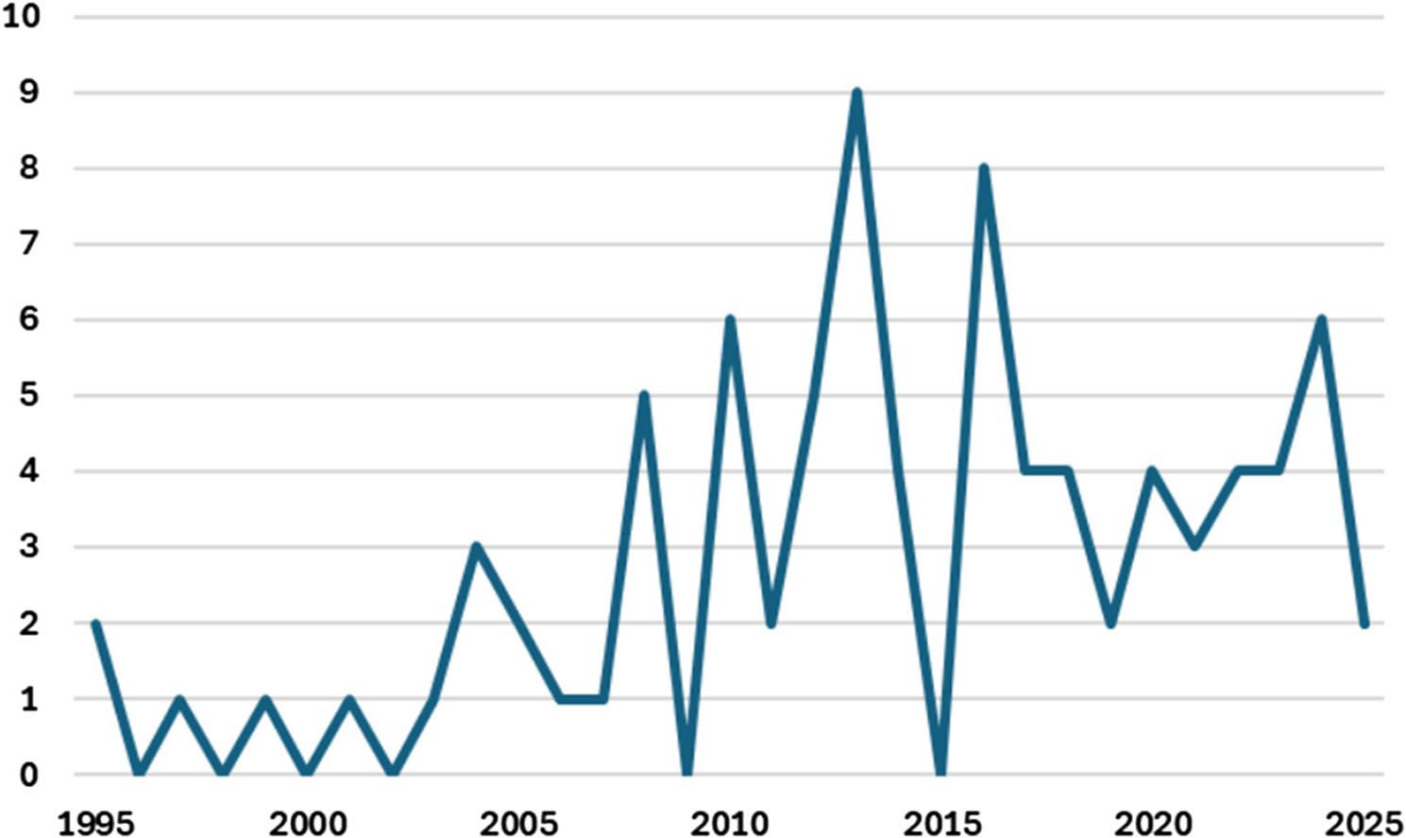
Number of records by publication year

**Fig. 3:**
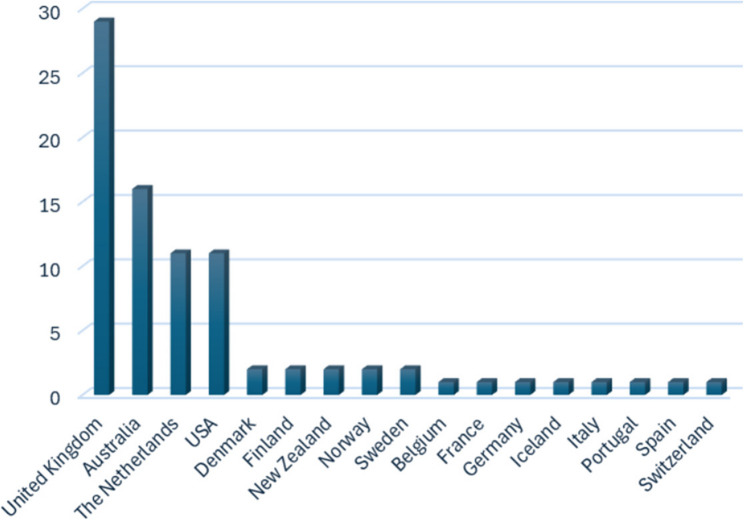
Number of records by country of origin

**Fig. 4:**
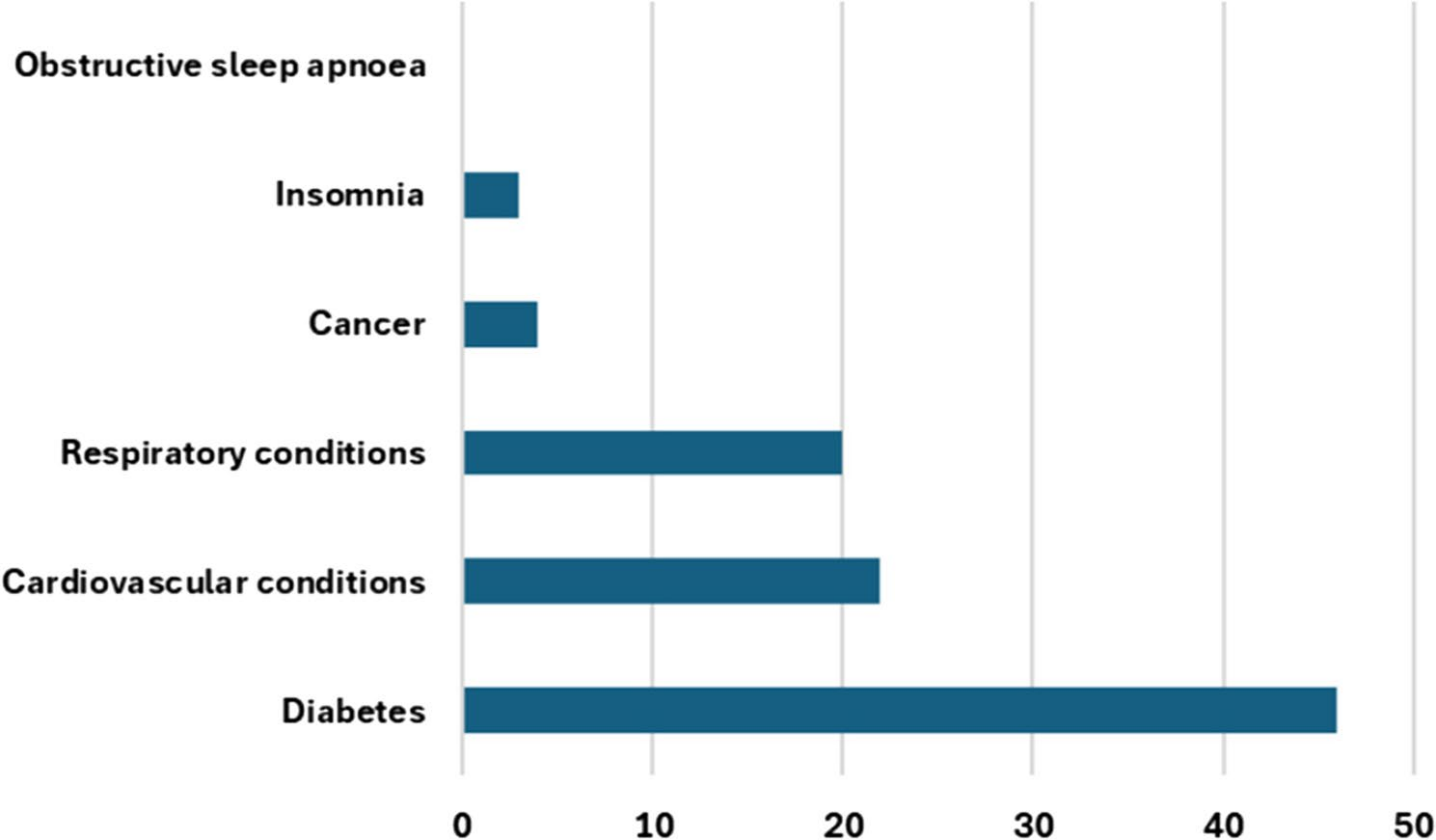
Number of records by chronic condition studied

Fifty-seven records contained data regarding both barriers and facilitators to implementation, 14 only reported facilitators, and 14 only reported barriers. Publications on the topic peaked in 2013 when nine records were published (Fig. 2). Records were conducted in 17 countries, with 34% (29/86) from in the UK, 20% (16/85) from Australia, 13% (11/85) from The Netherlands, and 13% (11/85) from the USA (Fig. 3). Diabetes was the most studied condition (46/85). Three records relate to insomnia (4%) and no records related to OSA were included in the review (Fig. 4). Details of individual records, including the classification of the nurse (if reported) and a description of the intervention, are listed in the study characteristics table (Table 2).

### Outcome of critical appraisal

All records included in the review were deemed to be of sufficient quality for inclusion by reviewers 1 and 2. Two records were eligible at full text screening but were found to be of insufficient quality at critical appraisal. Both records lacked detail about the analytical approach. Also, one did not provide quotes to support the qualitative findings, and in the other the lead researcher was also a clinician in one of two general practices in the study and did not address the influence that this had on the research. Critical appraisal was unable to be completed on two records. One, a conference abstract with no associated full text record, was excluded as it lacked detail about methodology and contributed very little data to the review. The other was missing detail about methodology but contained a significant amount of data pertinent to the review [96]. The record was published in 1997, prior to the advent of implementation science, and therefore did not adhere to current reporting guidelines. As the record was otherwise deemed to be of sufficient quality, it was agreed to include this record in the review.

### Development of a determinant framework specific to the implementation of general practice nurse-delivered models of care for the management of chronic conditions

Extracted data was mapped to the primary and secondary themes of the Lau framework. The Lau framework consists of four primary themes that represent contexts in which barriers and facilitators can influence the implementation of complex interventions in primary care: external context, organisation, professional, and intervention. Data relevant to all four primary themes and 19 of 22 secondary themes were identified.

**Fig. 5:**
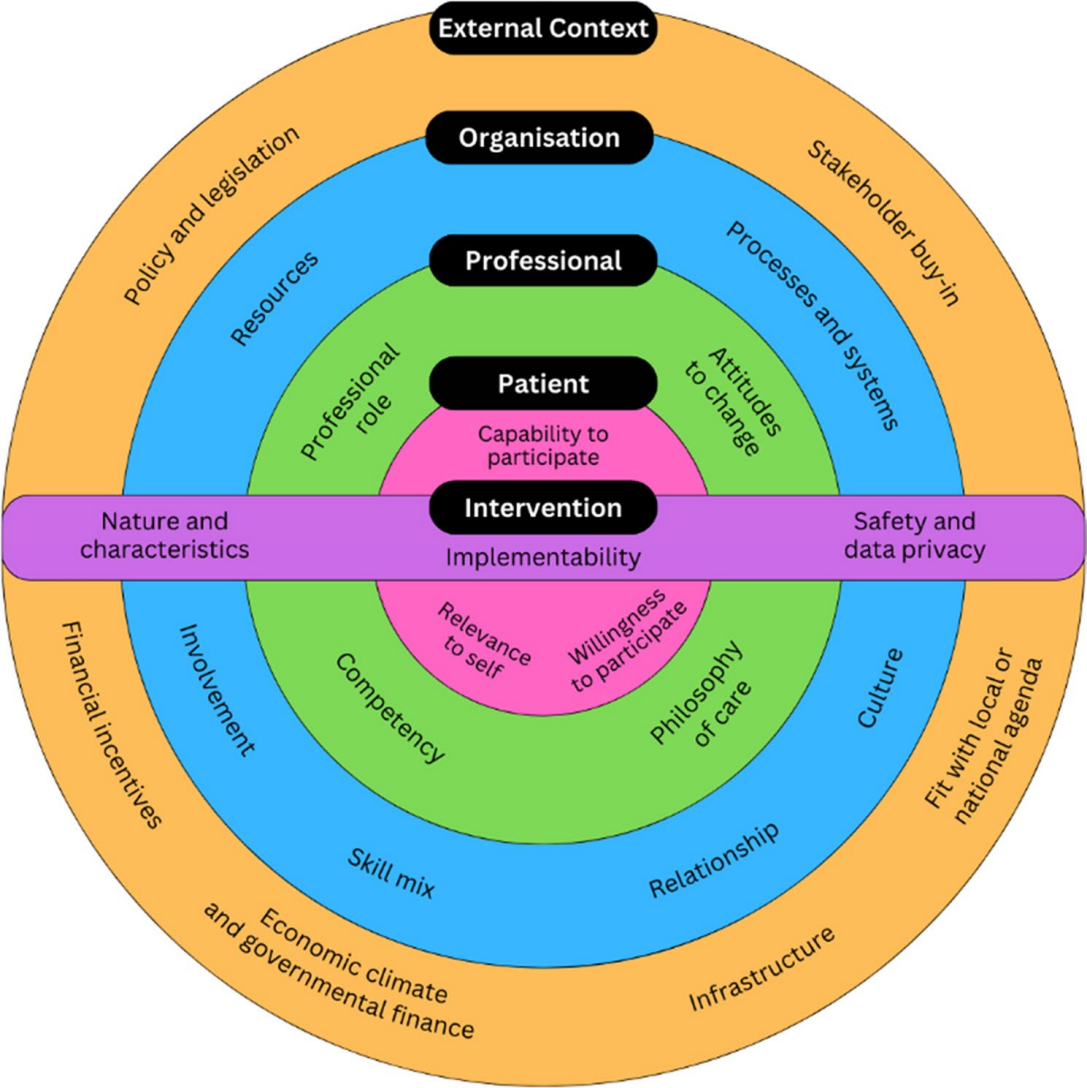
Factors influencing the Implementation of models of care for Chronic Conditions in General Practice (FICC-GP) determinant framework

During data extraction, it was identified that there was data that did not fit within the themes of the Lau framework in a way that adequately represented the meaning of the data. These data were patient-related factors that influenced the implementation of a model of care. To categorise and adequately represent these findings in this review, an additional primary theme (‘Patient’) and sub-themes (‘Capability to participate’, Relevance to self’, and ‘Willingness to participate’) were formed. By adding this primary theme and sub-themes, and by removing the three sub-themes that did not relate to this review (‘public awareness’, ‘dominant paradigm’, and ‘technology advances’), an amended determinant framework specific to the implementation of general practice nurse-delivered models of care for the management of chronic conditions was developed: ‘Factors influencing the Implementation of models of care for Chronic Conditions in General Practice’ (FICC-GP) (Fig. 5). This framework was used to synthesise the data and structure the narrative presentation of findings in this review. The barriers and facilitators identified during data extraction are presented in a data synthesis table (Table 3).

### External context

#### Policy and legislation

A lack of legislation and legal frameworks was found to limit the development of sustainable ongoing services [120, 137]. Recognition of the value of the intervention by the government facilitated implementation in one study [120] while a lack of support from the government was motivation for practices to remain focused on delivering the intervention in another, however, in this study the general practices involved were considered to be “early-adopters” [131].

#### Stakeholder buy-in

Recognition of the value of the model of care by the governing organisation and alignment with the values of the organisation facilitated implementation [98], whereas a lack of funding for the intervention by insurance companies was a barrier [74].

#### Fit with local or national agenda

Considered timing of implementation was found to be important with one study reporting difficulty implementing a model of care for diabetes management during a restructure of primary care services [133]. Another encountered challenges when changes to the nursing profession were occurring, which cast doubt on the introduction of a new nursing role [77]. The COVID-19 pandemic also impacted implementation, limiting face-to-face care and physical assessments [117], and increasing the workloads of general practice staff [148].

#### Infrastructure

Access to adequate information technology was found to facilitate implementation [120] while lack of a centralised information technology system was a barrier [78, 98].

#### Economic climate and governmental financing

Funding that was consistent [78] and able to be used to employ nurses [48, 83, 100] facilitated the implementation of models of care, whereas funding that was insufficient [72, 78, 120], incentivised short consultation times [72] or did not support care delivery by a multi-disciplinary team [120, 137] was a barrier. Six studies reported a lack of funding for nurse-delivered care, three from Australia [79, 100, 108], and one each from Switzerland [121], Sweden [83], and the Netherlands [88], and another Australian study reported a lack of funding for the intervention itself (chronic obstructive pulmonary disease management) [87].

#### Financial incentives

Renumeration for nurses that reflected the increased responsibilities associated with a model of care facilitated implementation [129], whereas inadequate renumeration was a barrier [79]. Insufficient financial incentives was also a barrier to a practice delivering an intervention [72, 144], however, in one study, staff were not engaged with a model of care even though they were provided adequate reimbursement as it did not meet the needs of patients [127].

### Organisation

#### Resources

Lack of time to deliver a model of care within the context of an already busy workload was the most commonly reported factor in the review. Lack of time was identified as a barrier in 24 studies [69, 70, 72, 73, 75, 79, 81–83, 87, 94, 97, 99–101, 125, 127–129, 132, 144, 147, 148]. This issue was reported in studies from five countries, the United Kingdom (UK) (12 records), Australia (6 records), The Netherlands (2 records), Norway (2 records), and the United States of America (US) (2 records). Having limited time to commit to implementation made the establishment of models of care challenging [79, 90, 137] as staff lacked the time to discuss the plan for implementation and practice using the intervention [75, 119], seek support from specialist staff [125], and attend training [82, 90, 116, 127, 134]. Protected time to deliver an intervention and having a dedicated staff member to deliver the intervention were found to be a facilitators to implementation [81, 97, 129, 138, 141], however finding the time to implement models of care was difficult even with adequate funding [118, 133]. Having sufficient staff involved in the model of care was a facilitator to implementation in six studies [69, 72, 74, 81, 99, 128], while insufficient staffing was a barrier, limiting backfill for nurses to attend training [70] and reducing the capacity of trained nurses to deliver the intervention [72].

It was difficult for practices to engage with a model of care if they incurred a financial cost, such as the cost of training staff [81, 112], particularly if they didn’t have established funding [121]. Lack of physical space [69, 71, 72, 79, 90, 118, 121], inefficient information technology support [81, 110, 112, 132] and a lack of quality equipment [70, 72, 82] were also barriers to implementation, whereas access to existing resources [98, 120], sufficient equipment [74], and ongoing information technology support [74] were facilitators to implementation of models of care.

#### Processes and systems

Appointment scheduling that provided sufficient time with the patient allowed for development of the nurse-patient relationship [103, 124], patient engagement [50, 83, 93, 99, 100], and the delivery of patient-centred care [108, 128, 148], however long appointments were considered expensive to deliver [148]. Conversely, short nursing appointments were a barrier to patient engagement [127], the establishment of trust between the nurse and the patient [127], and effective delivery of an intervention [68, 119]. Continuity of nursing staff improved nurse and patient engagement with a model of care [90, 108, 128], trust and rapport in the nurse-patient relationship [93, 142], and facilitated the delivery of patient-centred care [142].

#### Culture

A positive workplace culture [75] and regular team meetings [96, 105] were facilitators to implementation. Frequent changes to practice priorities were found to be a barrier [90].

#### Relationship

Relationships between practice team members and between the clinician and the patient influenced implementation. A collaborative and trusting nurse-doctor relationship [48, 104], GP confidence in the nurse [108], and respect and trust between team members [75, 108] facilitated implementation. Effective communication between the GP and nurse facilitated both implementation [100] and a collaborative approach to care [148]. Shared decision making [73], and trust [48] between the patient and the clinician facilitated patient engagement, and ongoing support from clinicians improved both patient engagement [111, 122] and adherence to treatment [101].

A relationship between general practice and specialist care was also found to facilitate implementation [94]. Having support from condition-specific specialist staff facilitated education of practice staff [106] and improved their confidence in intervention delivery [125, 128, 129, 143]. Conversely, a poor relationship with specialist services limited access to specialist support when needed [112] and limited a nurse’s capacity to ascertain their competence in intervention delivery [125].

#### Skill mix

Clear roles for team members [74, 78], agreement between team members about accountability for the intervention [75], and shared responsibility for the delivery of interventions [50, 75, 108] facilitated implementation, whereas uncertainty about the roles of team members was found to be a barrier [75, 78, 134, 148].

#### Involvement

Involvement of the broader practice team in a model of care was a facilitator to implementation [103, 128, 129]. Demonstration of teamwork between practice staff instilled confidence in patients [50, 93, 99], and increased motivation [131] and confidence in nurses to deliver an intervention [82], whereas lack of teamwork was a barrier to implementation [72, 78, 141]. General practitioner support of a model of care improved the confidence of nurses to deliver a model of care [79, 82, 125, 129] and facilitated patient confidence in an intervention [50, 87, 122]. A lack of support from GPs had significant impact on the involvement of nurses in models of care. It reduced their capacity to be involved in a model of care [81, 132], their confidence in care delivery [82, 125], and their interest in a model of care [69], and also instilled feelings of isolation in nurses [90]. Similarly, lack of peer nursing support reduced nurse engagement in models of care [74] and confidence in nurses [82]. Working in isolation also made it difficult for nurses to obtain support from their peers [82] and ascertain their competence in the delivery of an intervention [90, 129].

### Professional

#### Professional role

Lack of a defined scope of nursing practice was found to be a barrier to implementation [69, 77, 96], particularly in the management of diabetes. General practice nurses implementing new models of care for type 2 diabetes management were concerned about working outside of their scope of practice [128, 133] and feared that having an unclear scope of practice could impact their professional indemnity insurance [129]. Lack of clarity about the scope of practice of nurses was found to be a barrier to patient engagement [111] and limited general practice nurses from taking on additional responsibilities, with GPs reluctant to delegate work to nurses if they were unsure about the scope of practice or capabilities of the nurse [75, 79]. However, a GP’s confidence in a nurse’s knowledge and skills facilitated delegation of responsibilities from GPs to nurses [75]. Other factors limiting the delegation of responsibilities from GPs to nurses were GPs wanting to provide care themselves [90], insufficient workloads [121], fear of losing control [121], and difficulty accepting the cultural shift from a traditional hierarchical relationship to nurse-delivered models of care [121].

#### Attitudes to change

Staff interest and confidence in an intervention were found to be facilitators to implementation [72, 120]. Staff who believed in the value of the intervention [81], that it would be successful [98] and improve patient outcomes [116, 148], were more likely to be engaged with a model of care. However, staff who were uncertain about the effectiveness and benefits [74, 96, 143], appropriateness [75, 110, 144], cost-effectiveness [74, 132], or scope of the model of care [86, 116], or were concerned about how the model would work with competing demands [70, 87, 98, 118, 137, 141], were more reluctant to change their behaviour to accommodate new models of care.

#### Philosophy of care

Approaches to care delivery that prioritised the needs of the patients, such as person-centred [68, 70, 83, 93, 99, 100, 108, 119, 124, 142], empathetic [104, 124], and holistic [93] care improved patient engagement however misalignment between the patient and health professional values and priorities made implementation more difficult for staff [72, 109, 114, 127].

#### Competency

Nurses who lacked knowledge also lacked confidence [125] and had reduced capacity to deliver an intervention [112, 134, 141]. Lack of knowledge also limited a nurse’s ability to provide culturally relevant care [141], use guidelines [85, 144], and develop relationships with external organisations [77]. GPs who lacked knowledge about the model of care found it more difficult to deliver the care [136] and support nurses in intervention delivery [82, 129], and patients who were aware or concerned that staff lacked competence were less likely to engage [74, 111].

Receiving insufficient training limited the capacity [72, 90] and confidence [74, 82, 83, 103, 110, 133] of practice staff to deliver an intervention. Whereas staff that received sufficient training were more likely to be confident in intervention delivery [79–81, 88, 101, 112] and aware of the value of the model of care [116], and nurses were more willing to accept accountability for care [129]. Training that is ongoing [82, 103, 129, 147], supplemented with practical experience [129, 143, 144], and delivered to the entire practice team improved confidence [82, 103, 129], and facilitated a team approach to the implementation of models of care [70, 81, 121].

### Intervention

#### Nature and characteristics

Interventions that were compatible with existing work practices [68, 70, 74, 98, 116, 120], and had structured [70, 74, 78, 88, 99, 108, 109, 113, 119, 132, 135, 136, 138] and adaptable [40, 48, 74, 127] resources were easier for practices to implement. Structured protocols were found to facilitate patient confidence in the delivery of care by nurses [111, 136], increase the knowledge, skills, and confidence of nurses [83, 136], and reduce the frequency of appointments [136]. Compatibility with existing practice software was a facilitator to implementation [70, 72, 86, 88, 112], whereas lack of integration with practice software was considered a barrier [74, 98, 113, 121]. Interventions that were complex [72, 121] and time consuming [82, 137] were also challenging for practices to implement.

General practice staff were more likely to be engaged with models of care that offered clear evidence of benefits to patients [90, 96, 98, 109, 112, 120, 122, 125], capability to track patient progress [86, 137], and tangible benefits for practice staff [131], such as reducing GP workload [101, 108] and adding value to the role of the nurse [88].

Patient engagement was limited in models of care that were time consuming [74], too demanding [97, 101], delivered over a long period of time [142], used unfamiliar terminology [68, 110, 127], lacked immediate tangible results [134], and used resources that were not able to be adapted to meet their individual need [70, 86, 141]. Whereas models of care that provided tangible results [99, 122], demonstrated evidence of benefit [142], and offered opportunities for patients to connect with others [48, 74, 142] were associated with ongoing patient motivation and engagement.

#### Implementability

In some studies, general practice staff perceived the implementation of models of care as time consuming [48, 69, 110, 137] and a burden when already dealing with busy workloads [99, 128]. In one study of the implementation of nurse-delivered telemedicine for the management of hypertension, the timing of implementation proved to be important [98]. A delay in commencing implementation resulted in a loss of time allocated to the intervention, a reduction in staff enthusiasm about implementing the model of care, and a loss of staff knowledge. However, once implementation commenced and practice staff realised that minimal time was required for implementation, enthusiasm about the implementation process was regained.

#### Safety and data privacy

Lack of software security was a barrier to the use of intervention software by patients [74].

### Patients

#### Capability to participate

Social determinants of health influenced the capability of patients to engage with a model of care. Having a primary language that was different to the language used to deliver the intervention [74, 88, 112, 132, 134, 141], illiteracy or limited literacy [74, 134, 146], poor health literacy [88, 141], or technology illiteracy [112, 136] made participating in an intervention more challenging for patients. Having limited time to dedicate to the intervention [86, 122, 139], competing caring responsibilities [48, 127], financial constraints (low socio-economic status [88, 134], and no access to the resources needed to participate [86, 88, 112, 134, 136, 139]) also made it more difficult for patients to engage with a model of care. Health status also influenced patient capability, with multi-morbidity [88, 132], physical ill health [95, 116], competing health priorities [48, 68, 81, 100, 101, 118, 119], pain [112], and visual impairment [97, 112] identified as barriers. Patients with good self-management skills [103], support from family [122, 142], and self-reported stable conditions [108] were more capable of participating in models of care.

#### Relevance to self

A patient’s beliefs about the relevance of the intervention to their condition or circumstance influenced engagement with a model of care. Believing that their condition was mild and as such doesn’t require treatment [68, 74, 112], untreatable [103], too chronic [112], or too mild [68, 74, 112] reduced patient engagement with an intervention. Also, patients who considered an intervention to be low priority [128] or not appropriate for them due to being older [68], they were less likely to engage, whereas those that believed that the intervention would be useful [112] or needed [97], or that it would mean that they would receive treatment faster [111] were more engaged.

#### Willingness to participate

Lack of patient engagement was a barrier to implementation [70, 92, 97, 133]. Non-attendance at appointments [74, 75, 128, 137, 141], non-adherence to recommended treatment [74, 92], and unreadiness to make change [81, 124, 128] made intervention delivery challenging for practice staff. Fear, of the intervention [75, 127] and of social stigma related to the intervention [72, 127], peer pressure from others to not participate [134], considering the intervention to be an unwelcome reminder of their health issues [112], and concern about burdening their already busy GP [109] made some patients reluctant to engage with an intervention. However, patients were motivated to engage with a model of care if they believed that it would free up health professionals’ time [50, 108, 112] or improve their health status [100, 122, 142].

## Discussion

This review was conducted to synthesise existing knowledge about the factors influencing the implementation of general practice nurse-delivered models of care for the management of chronic conditions and has generated theory-driven evidence and a determinant framework that can be used to guide future work. While the barriers and facilitators presented in the findings of this review can, and arguably should, be used pragmatically when developing new models of care, it is the interaction of these factors that brings greater meaning to the findings of this review. This review paints a picture of general practice as a complex and busy environment, with the practice team trying to work together to deliver quality chronic condition management, but struggling with the competing demands of general practice, uncertainty about their roles, and limited time to dedicate to the implementation of new models of care. This is consistent with the finding from Lau et al. that the implementing change in primary care is complex [56].

This review clearly demonstrates that the implementation of models of care for chronic conditions in general practice is not performed in isolation by any one individual or professional group, that teamwork is a facilitator to implementation, and that the role of the GP and the general practice nurse are intrinsically linked. This review finds that, while nurses are often delivering the intervention, they benefit from the support of other health professionals to feel confident and competent in intervention delivery. Most significantly, nurses rely on GPs for support, confidence, and trust, but they also benefit from support from the broader practice team, including other nurses, and from external specialist staff. In light of these findings, we recommend that the term ‘nurse-delivered’ be used to describe a model of care if nurses are the clinician primarily delivering the intervention and the term ‘general practice-delivered’ be used when describing models of care that are delivered collaboratively by a general practice team. We also recommend that the term ‘nurse-led’ is not used to describe models of care or interventions delivered in the general practice setting as it does not reflect the team-approach to care that is evident within this review.

It was identified in this review that a lack of clarity about the roles of team members in models of care made implementation challenging and that uncertainty about the scope of practice of general practice nurses was a barrier to nurses having a greater role in models of care. Of note, GPs reported a reluctance to delegate work to general practice nurses if they were unsure about the nurse’s scope of practice or capabilities. These findings are echoed in a systematic review published in 2021 which explored the factors influencing the implementation of the nursing role into primary care settings [150]. This review found that uncertainty about roles, a lack of awareness of a nurse’s scope of practice, and a lack of trust in the capabilities of the nurse were barriers to delegation of responsibilities from GPs to nurses. However, it was also identified that having good experiences collaborating with nurses made GPs more aware of the scope of practice of nurses, and nurses being satisfied with their scope of practice is a facilitator to implementing a nursing role in primary care. Similarly, a review exploring the barriers and facilitators to teamwork between GPs and general practice nurses found that a lack of clarity or confusion about the scope of practice and role of nurses was a barrier to collaborative practice and contributed to an erosion of trust in the competencies of nurses [151]. In light of these findings, we recommend that the roles of team members are clearly defined and communicated to all staff when designing and implementing models of care in general practice to facilitate a shared understanding of the roles and responsibilities of team members. This is also a guiding principle of the CCM, with defined roles for team members considered to be an essential component of effective and efficient chronic condition care [57]. We also recommend that governments and professional regulatory bodies provide guidance and support to general practice nurses and GPs to better understand, define, and communicate the scope of practice of general practice nurses to support the delegation of responsibilities from GPs to nurses. In turn, this could lead to reducing demand in general practice by easing workload pressures for GPs, and increasing the scope of practice of general practice nurses [151]. An example of how this could be applied to sleep health care is in the management of chronic insomnia. Randomised controlled trials have demonstrated that general practice nurses can be trained to effectively treat patients with chronic insomnia by delivering a non-pharmacological treatment (cognitive behavioural therapy for insomnia) [152]. If the scope of this new nursing role in insomnia management was well defined, documented, and communicated to general practice nurses and GPs, GPs could then confidently delegate the management of chronic insomnia to general practice nurses, thus sharing the often frequent and lengthy appointments that they have with patients to discuss the management of chronic insomnia with nurses [27, 40, 153]. We acknowledge that there has been some progress towards defining the role of general practice nurses, however there still is more to be done to support general practice nurses to work to their full scope of practice. A comprehensive core capabilities framework for primary care and general practice nurses was released by the NHS in England in 2021. This document clearly outlines the roles and the expected capabilities of specific nursing classifications in England, and this could be a model used to define the role of the general practice nurse in other countries [154]. In Australia, a government-initiated review of the scope of practice of the primary care workforce has recently been completed [155]. Instigated in response to workforce shortages and increasing demand in Australian primary care, this review recommends increased educational opportunities for primary health care professionals, changes to general practice funding that promote multidisciplinary team care, and support for the establishment of nurse-led models of care in general practice.

Collaboration between primary and tertiary health care was identified as a facilitator to the implementation of models of care in this review. As specialist generalists, GPs and general practice nurses provide care for many different chronic conditions and as such cannot be experts in every chronic condition [156, 157]. This review shows that access to condition-specific specialists provides practice staff with the support, education, and confidence to deliver a condition-specific intervention. While there are clear benefits to collaboration between specialist care and general practice, these relationships can be difficult to establish and maintain, with relationships dependent on personal motivation, renumeration, and clinical guidelines that are conducive to collaborative working [158]. Therefore, we recommend that administrators or researchers facilitate relationships between general practice staff and relevant local tertiary specialist services when implementing condition-specific models of care, ensure that renumeration for the activities required for a collaborative relationship is available, and that models of care are embedded with processes that facilitate collaborative relationships between specialist care and general practice. Examples of how these recommendations could be implemented in sleep health care are formalising a relationship between tertiary public sleep services and general practices that elect to deliver OSA assessment and management, offering renumeration to specialist sleep services for providing support to general practice teams, and providing guidelines to general practice teams and specialist sleep services about how and when to seek and provide support.

Compatibility of the model of care with existing workflows and resources was identified as a facilitator to practice staff engagement. Practice staff reported difficulty finding the time to trial an intervention prior to implementation, and that implementation activities added to an already busy workload. As such, it is reasonable to assume that models of care that require considerable adaptions to existing work processes would be difficult for practices to implement. Understanding context when planning and implementing interventions is one of the foundations of implementation science, as without an understanding of the specific complexities of the context, widespread implementation will be challenging [159]. Therefore, we recommend that models of care are designed collaboratively with individuals who have first-hand knowledge of the context using participatory action research or consumer co-design approaches [159–161]. This could be applied to sleep health care by co-designing models of care for OSA and chronic insomnia with individuals who have experience in general practice, such as general practice staff and administrators. A collaborative approach to design and implementation with individuals who have lived experience and knowledge of the context would facilitate compatibility of the model of care with existing general practice work processes and resources, thus making delivery of models of care more feasible in a busy general practice.

Insufficient, inflexible, and delayed funding is a barrier to the establishment and delivery of models of care. Funding models that do not support team-based care, cannot be used for nurses to deliver care, or financially incentivise short appointments, such as time-tiered GP attendance items in the Australian Government Medicare Benefits Schedule [162], limit implementation of general practice-delivered models of care for the management of chronic conditions. Adequate time with the patient was found to facilitate an effective and trusting nurse-patient relationship. This is the foundation for shared decision making and person-centred care, and the essence of effective chronic condition management and principles of the CCM [57]. Without sufficient time with patients, nurses are unable to develop a therapeutic relationship that can meaningfully contribute to chronic condition management. As such, to facilitate the delivery of models of care, funding models should be structured to align with the best practice principles of the CCM by rewarding the delivery of quality, patient-centred care, and the length and frequency of appointments and the clinician delivering care should be at the discretion of the practice to allow practices to tailor approaches to chronic condition management to meet individual patient need.

Evidence of the benefits of the model of care was a facilitator to engagement by practice staff and patients, with financial incentives not necessarily enough to motivate staff. Patients and practice staff are motivated to engage with an intervention if they believe that it has benefit to patients or the practice. This finding is supported by theory, with the COM-B model of behaviour change recognising that with knowledge, capability, and opportunity, individuals can be motivated to make behaviour change [163]. As such, we recommend that evidence-based anticipated benefits of a model of care are communicated to practice staff to enhance practice staff engagement and equip staff with the knowledge and confidence to educate patients about the relevance of the intervention to their individual circumstance. An example of this is providing education to general practice clinicians about OSA and the use and benefits of continuous positive airway pressure (CPAP), so that they have the confidence and knowledge to discuss CPAP with patients and troubleshoot CPAP issues, if and when they arise [164].

This review clearly highlights that patients have influence on the implementation of models of care. Consideration of these patient-related factors is essential as, without patient engagement, any efforts to deliver a model of care will be in vain. The patient-related factors identified in this review also align with the COM-B model of behaviour change [163]. A patient’s health and social influences (capability), their perception of the relevance of the intervention to their circumstances (motivation), and their access to the resources needed to participate (opportunity) were identified as influencing a patient’s capacity to engage with an intervention. As such, we recommend that patient-related capabilities (such as literacy and access to resources), motivation (ensuring that models of care meet the needs of the intended population and that patients understand the relevance of the intervention to their condition or circumstance) and opportunity (ensuring that interventions are delivered at a location and at a time that is suitable for patients) are considered when designing a new model of care. Actively involving patients in the design process (co-design) of models of care would also contribute to ensuring that models of care for chronic condition management are patient-centred, accessible, and acceptable to patients [165].

*Value of the findings of this review beyond nurse-delivered models of care for sleep health care*.

Whilst this review was designed to inform the implementation of nurse-delivered models of care for the management of chronic conditions and sleep disorders, we believe that the findings have broader relevance to team-based care within general practice beyond the scope of nursing, chronic conditions, and sleep health care. The findings and recommendations of this review can be applied to the design and implementation of models of care in general practice, irrespective of the condition, chronicity of the condition, or the clinician delivering the intervention. We suggest that the amended determinant framework developed within this review (FICC-GP) be tested as a framework for future studies exploring the implementation of models of care in general practice, ideally in conjunction with consumers (general practice staff and patients) using co-design principles, and adapted as appropriate.

### Recommendations for practice

In summary, to facilitate the implementation of general practice-nurse delivered models of care it is recommended that:

1. The term ‘nurse-led’ is not used to describe interventions or models of care that are delivered in general practice as it does not reflect the team-based approach to general practice care.
2. The roles of team members in models of care are clearly defined and communicated with the entire general practice team to facilitate a team-based approach to care.
3. Support is provided to general practice nurses and GPs to understand and define the scope of practice of general practice nurses to increase GP confidence in delegating responsibilities to nurses.
4. Relationships between general practice teams and specialist care services are facilitated to ensure that general practice teams have support when delivering condition-specific models of care.
5. General practice staff are provided with evidence about the anticipated benefits of a model of care so that they can ascertain which of their patients would gain benefit from the model of care and motivate patients to engage with treatment.
6. Models of care are designed with general practice staff, administrators, and patients to optimise compatibility with existing processes and patient needs.

### Strengths and limitations of the review

To the best of our knowledge, this is the first systematic review on this topic. To identify all records on this topic, a mixed-methods review was conducted with no start date. This approach has resulted in the inclusion of studies employing various methodologies and spanning four decades to inform our recommendations. Whilst every effort was made to identify all records on this topic, we acknowledge that not all reports may have been discovered, however, with 85 records and minimal contradiction of findings, we believe that any missing literature would be unlikely to change the findings and recommendations. We also appreciate that the findings of this review are based only on literature related to the chronic conditions chosen for this review, and with additional or different chronic conditions the findings could be different. However, as these priority chronic conditions of the World Health Organization accounted for six of the top eight chronic conditions causing death in high income countries in 2021, it was deemed suitable to include these conditions and the sleep conditions of interest in this review [4]. It is also acknowledged that as only countries with a developed economy were included in this review, this limits the generalisability of the review for countries with transitioning and developing economies.

All stages of the review process were conducted with integrity and rigour, following JBI principles for mixed methods reviews [52] and the protocol was peer-reviewed and published [59]. Whilst only 20% of coding was checked by a second reviewer, there was substantial agreement of the meaning of the data in the coding that was checked, and extensive discussion occurred between reviewers throughout the review process. Reviewers and authors agree on the key findings, adapted framework, and recommendations in this paper.

### Implications for future research

It was intended that implementation strategies and nurse classification would be considered during data analysis however this was not possible as nurse classification was reported in only 24% of records, and specific implementation strategies were often not reported. While the outcome of the models of care was not the focus of this review, without detail about implementation strategies, any analysis of the association between implementation strategies and the outcome of models of care is not possible. Therefore, we suggest that greater emphasis is placed on describing implementation strategies and reporting nurse classification so that that these details can be considered in future analyses.

## Conclusion

This systematic review has synthesised the learnings of 85 studies to create high-quality theory-driven evidence, an amended determinant framework, and recommendations for future practice that can be used to inform the implementation of general practice-delivered models of care for chronic sleep disorders and chronic conditions more broadly. It is envisaged that if the recommendations made in this review are adopted by policy makers, clinicians, and researchers they would experience greater success when implementing new models of care in general practice, thus saving time and resources, and offering greater continuity of care for patients.

This review highlights the importance of considering patients when designing and implementing models of care. Facilitating patient involvement in their own health care and patient-centred care has been recognised as an essential component of chronic condition management for many years, and yet this review demonstrates that many models of care and funding models for chronic conditions do not consider the capabilities or motivations of patients in their design. Positioning patients at the centre of models of care, by involving general practice consumers in co-design processes and considering patient-related factors when designing, implementing, and evaluating models of care, will be a step forward to ensuring that models of care for chronic condition management meet the needs of the recipients of the care.

## Supplementary Information

Additional file 1. Database search strategies

Additional file 2. Data extraction table

## Data Availability

The datasets supporting the conclusions of this article are included within the article and its additional files.

## Abbreviations

CBTi: cognitive behavioural therapy for insomnia
CCM: Chronic Care Model
CINAHL: Cumulative Index to Nursing and Allied Health Literature
COPD: chronic obstructive pulmonary disease
CPAP: continuous positive airway pressure
EBSCO: Elton B. Stephens CO (company)
FICC-GP: Factors influencing the Implementation of models of care for Chronic Conditions in General Practice
GP: general practitioner
JBI: Joanna Briggs Institute
OSA: obstructive sleep apnea
PRISMA: Preferred Reporting Items for Systematic Reviews and Meta–Analyses
UK: United Kingdom
USA: United States of America
