# Supplementary material for: Factors influencing the implementation of general practice nurse-delivered models of care for chronic conditions: a mixed-methods systematic review to inform models of care for chronic sleep disorders
Source: BMC Prim Care. 2026 Feb 14;27:98. doi: 10.1186/s12875-025-03078-4 (PMC13011425; doi:10.1186/s12875-025-03078-4)
Supplement: Supplementary file 1 — Additional file 1. Database search strategies [file 12875_2025_3078_MOESM1_ESM.docx]

**Database search strategies**

All conducted on 2^nd^ June 2022 (no start date -2^nd^ June 2022) and 4^th^ July 2025 (2022 – current)

CINAHL

S23 S21 AND S22

S22 TI ( (Australia* OR Austria* OR Belgium OR Belgian OR Bulgaria* OR Canad* OR Croatia* OR Czechia* OR "Czech Republic" OR Denmark OR Danish OR Estonia* OR Finland OR Finnish OR France OR French OR German* OR Greece OR Greek OR Hungar* OR Iceland* OR Ireland OR Irish OR Japan* OR Italy OR Italian OR Latvia* OR Lithuania* OR Luxembourg* OR Malta OR Maltese OR Netherlands OR Dutch OR "New Zealand*" OR Norway OR Norwegian OR Poland OR Polish OR Portug* OR Romania* OR Slovakia* OR Sloveni* OR Spain OR Spanish OR Sweden OR Swedish OR Switzerland OR Swiss OR "United Kingdom" OR UK OR England OR English OR Scotland OR Scottish OR Wales OR Welsh OR "United States" OR USA) ) OR AB ( ( Australia* OR Austria* OR Belgium OR Belgian OR Bulgaria* OR Canad* OR Croatia* OR Czechia* OR "Czech Republic" OR Denmark OR Danish OR Estonia* OR Finland OR Finnish OR France OR French OR German* OR Greece OR Greek OR Hungar* OR Iceland* OR Ireland OR Irish OR Japan* OR Italy OR Italian OR Latvia* OR Lithuania* OR Luxembourg* OR Malta OR Maltese OR Netherlands OR Dutch OR "New Zealand*" OR Norway OR Norwegian OR Poland OR Polish OR Portug* OR Romania* OR Slovakia* OR Sloveni* OR Spain OR Spanish OR Sweden OR Swedish OR Switzerland OR Swiss OR "United Kingdom" OR UK OR England OR English OR Scotland OR Scottish OR Wales OR Welsh OR "United States" OR USA ) )

S21 S6 AND S20

S20 S7 OR S8 OR S9 OR S10 OR S11 OR S12 OR S13 OR S14 OR S15 OR S16 OR S17 OR S18 OR s19

S19 TI ( diabetes OR NIDDM OR IDDM ) OR AB ( diabetes OR NIDDM OR IDDM )

S18 (MH "Diabetes Mellitus")

S17 TI ( (cancer* OR carcinoma* OR neoplas* OR tumo#r* OR malignan* OR oncolog* OR leuk#emia* OR metasta* OR lymphoma* OR melanoma* ) ) OR AB ( (cancer* OR carcinoma* OR neoplas* OR tumo#r* OR malignan* OR oncolog* OR leuk#emia* OR metasta* OR adenoma* OR adenocarcinoma* OR adeno-carcinoma* OR lymphoma* OR melanoma* ) )

S16 TI ( hypertension OR "high blood pressure" OR "elevated blood pressure" OR htn OR hypertensive ) OR AB ( hypertension OR "high blood pressure" OR "elevated blood pressure" OR htn OR hypertensive )

Database - CINAHL 108,683

S15 (MH "Neoplasms+") OR (MH "Cancer Patients")

S14 (MH "Coronary Arteriosclerosis")

S13 (MH "Heart Failure")

S12 (MH "Hypertension")

S11 (MH "Asthma")

S10 (MH "Cardiovascular Diseases")

S9 (MH "Pulmonary Disease, Chronic Obstructive")

S8 (MH "Insomnia")

S7 (MH "Sleep Apnea, Obstructive") OR "sleep health"

S6 S3 AND S4 AND S5

S5 TI ( ( implementation OR evaluation* OR barrier* OR challenge* OR facilitat* OR feasib* OR enable* OR "process evaluation" or “implementation science”) ) OR AB ( ( implementation OR barrier* OR facilitat* OR feasib* OR enable* OR "process evaluation" OR “implementation science”) )

S4 TI ( (nurse-led OR nurse-manag* OR nurse-delivered OR (nurse* N5 (led OR manage* OR program* OR model* OR provision OR intervention* OR service OR system* OR care OR healthcare OR deliver* OR treat* OR pathway))) ) OR AB ( (nurse-led OR nurse-manag* OR nurse-delivered OR (nurse N5 (led OR manage* OR program* OR model* OR provision OR intervention* OR service OR system* OR care OR healthcare OR deliver* OR Treat* OR pathway* ))) )

S3 S1 OR S2

S2 TI ( ( ( ( family OR general ) N2 ( physician* OR doctor* OR practitioner* OR medicine OR practice* ) ) OR GP* OR "primary health provider*") ) OR AB ( ( ( ( family OR general ) N2 ( physician* OR doctor* OR practitioner* OR medicine OR practice* ) ) OR GP* OR "primary health provider*") )

S1 (MH "Physicians, Family")

*2^nd^ June 2022: records identified: 152*

*4^th^ July 2025: records identified: 38*

Scopus

TITLE-ABS-KEY ( ( ( ( family OR general ) W/2 ( physician* OR doctor* OR practitioner* OR medicine OR practice* ) ) OR gp* OR "primary health provider*" ) AND ( nurse-led OR nurse-manag* OR nurse-delivered OR ( nurs* AND ( "models of care" OR "model of care" OR "care model" ) ) OR ( nurse W/5 ( led OR manage* OR program* OR model* OR provision OR intervention* OR service OR system* OR care OR healthcare OR deliver* OR treat* OR pathway* ) ) ) AND ( "heart failure" OR copd OR coad OR "chronic obstructive pulmonary disease" OR "coronary artery disease" OR "chronic obstructive airways disease" OR asthma OR "cardiovascular disease" OR "congestive heart failure" OR "type 2 diabetes" OR niddm OR diabetes OR iddm OR "type 1 diabetes" OR cancer* OR carcinoma* OR neoplas* OR malignan* OR oncolog* OR leukemia* OR metasta* OR lymphoma* OR melanoma* ) AND ( implementation OR barrier* OR facilitat* OR feasib* OR enable* OR "process evaluation" OR dissemination OR "implementation science" ) AND (Australia* OR Austria* OR Belgium OR Belgian OR Bulgaria* OR Canad* OR Croatia* OR Czechia* OR "Czech Republic" OR Denmark OR Danish OR Estonia* OR Finland OR Finnish OR France OR French OR German* OR Greece OR Greek OR Hungar* OR Iceland* OR Ireland OR Irish OR Japan* OR Italy OR Italian OR Latvia* OR Lithuania* OR Luxembourg* OR Malta OR Maltese OR Netherlands OR Dutch OR "New Zealand*" OR Norway OR Norwegian OR Poland OR Polish OR Portug* OR Romania* OR Slovakia* OR Sloveni* OR Spain OR Spanish OR Sweden OR Swedish OR Switzerland OR Swiss OR "United Kingdom" OR UK OR England OR English OR Scotland OR Scottish OR Wales OR Welsh OR "United States" OR USA) )

*2^nd^ June 2022: records identified: 334*

*4^th^ July 2025: records identified: 93*

Emcare

1. general practitioner/

2. family medicine/

3. ((family or general) adj3 (physician* or doctor* or practitioner* or medicine or practice*)).ti,ab,kf.

4. (GP* or "primary health provider*").ti,ab,kf.

5. or/1-4

6. (nurse-led or nurse-manag* or nurse-delivered or (nurse* adj6 (led or manage* or program* or model* or provision or intervention* or service or system* or care or healthcare or deliver* or treat* or pathway))).ti,ab,kf.

7. exp program development/

8. exp program evaluation/

9. exp Implementation Science/

10. (implementation or evaluation* or barrier* or challenge* or facilitat* or feasib* or enable* or "process evaluation" or "implementation science").ti,ab,kf.

11. or/7-10

12. 5 and 6 and 11

13. ("sleep health" or "obstructive sleep apnea" or "obstructive sleep apnoea").ti,ab,kf.

14. sleep disordered breathing/

15. (chronic adj6 insomnia).ti,ab,kf.

16. exp chronic obstructive lung disease/

17. (hypertension or "high blood pressure" or "elevated blood pressure" or htn).ti,ab,kf.

18. exp hypertension/

19. exp asthma/

20. exp cardiovascular disease/

21. ("heart failure" or copd or coad or "chronic obstructive pulmonary disease" or "chronic obstructive airways disease" or "coronary artery disease" or asthma or "cardiovascular disease").ti,ab,kf.

22. exp diabetes mellitus/

23. (diabetes or NIDDM or IDDM).ti,ab,kf.

24. exp malignant neoplasm/

25. (cancer* or carcinoma* or neoplas* or tumo#r* or malignan* or oncolog* or leuk#emia* or metasta* or lymphoma* or melanoma*).ti,ab,kf.

26. or/13-25

27. 12 and 26

28. australia/ or austria/ or belgium/ or bulgaria/ or canada/ or croatia/ or czechia/ or czech republic/ or denmark/ or estonia/ or finland/ or france/ or germany/ or greece/ or hungary/ or iceland/ or ireland/ or italy/ or japan/ or latvia/ or lithuania/ or luxembourg/ or malta/ or netherlands/ or new zealand/ or norway/ or poland/ or portugal/ or romania/ or slovakia/ or slovenia/ or spain/ or sweden/ or switzerland/ or united kingdom/ or england/ or scotland/ or wales/ or united states/

29. (greece or greek or hungar* or iceland* or ireland or irish or italy or italian or japan* or latvia* or lithuania* or luxembourg* or malta or maltese or netherlands or dutch or new zealand or new zealanders or NZ or norway or norwegian or poland or polish or portug* or romania* or slovakia* or slovenia* or spain or spanish or sweden or swedish or switzerland or swiss or united kingdom or UK or england or scotland or scottish or wales or welsh or ireland or irish or united states or USA or US).ti,ab,kf.

30. 28 or 29

31. 27 and 30

*2^nd^ June 2022: records identified: 222*

*4^th^ July 2025: records identified: 61*

Medline

1. General practice/ or Family practice/ or General Practitioners/ or Primary Health Care/ or Physicians, Family/

2. ((family or general) adj3 (physician* or doctor* or practitioner* or medicine or practice*)).tw,kf.

3. (GP* or "primary health provider*").tw,kf.

4. or/1-3

5. (nurse-led or nurse-manag* or nurse-delivered or (nurse* adj6 (led or manage* or program* or model* or provision or intervention* or service or system* or care or healthcare or deliver* or treat* or pathway))).tw,kf.

6. (implementation or evaluation* or barrier* or challenge* or facilitat* or feasib* or enable* or "process evaluation" or "implementation science").tw,kf.

7. Program Evaluation/ or Program Development/ or Implementation Science/

8. or/6-7

9. 4 and 5 and 8

10. ("sleep health" or "obstructive sleep apnea" or "obstructive sleep apnoea").tw,kf.

11. Sleep Apnea Syndromes/

12. (chronic adj6 insomnia).tw,kf.

13. exp Pulmonary Disease, Chronic Obstructive/ or exp Cardiovascular Diseases/ or exp Asthma/ or Hypertension/

14. (hypertension or "high blood pressure" or "elevated blood pressure").tw,kf.

15. ("heart failure" or copd or coad or "chronic obstructive pulmonary disease" or "chronic obstructive airways disease" or "coronary artery disease" or asthma or "cardiovascular disease").tw,kf.

16. exp Diabetes Mellitus/

17. (diabetes or NIDDM or IDDM).tw,kf.

18. exp Neoplasms/

19. (cancer* or carcinoma* or neoplas* or tumo#r* or malignan* or oncolog* or leuk#emia* or metasta* or lymphoma* or melanoma*).tw,kf.

20. or/10-19

21. 9 and 20

22. australia/ or austria/ or belgium/ or bulgaria/ or canada/ or croatia/ or czechia/ or czech republic/ or denmark/ or estonia/ or finland/ or france/ or germany/ or greece/ or hungary/ or iceland/ or ireland/ or italy/ or japan/ or latvia/ or lithuania/ or luxembourg/ or malta/ or netherlands/ or new zealand/ or norway/ or poland/ or portugal/ or romania/ or slovakia/ or slovenia/ or spain/ or sweden/ or switzerland/ or united kingdom/ or england/ or scotland/ or wales/ or united states/

23. (Australia* or austria* or belgium or belgian or bulgaria* or canad* or croatia* or czechia* or czech republic or denmark or danish or estonia* or finland or finnish or france or french or german or germany or greece or greek or hungar* or iceland* or ireland or irish or italy or italian or japan* or latvia* or lithuania* or luxembourg* or malta or maltese or netherlands or dutch or new zealand or new zealanders or NZ or norway or norwegian or poland or polish or portug* or romania* or slovakia* or slovenia* or spain or spanish or sweden or swedish or switzerland or swiss or united kingdom or UK or england or scotland or scottish or wales or welsh or ireland or irish or united states or USA or US).tw,kf.

24. or/22-23

25. 21 and 24

*2^nd^ June 2022: records identified: 468*

*4^th^ July 2025: records identified: 106*

Cochrane Library Trials

#1 MeSH descriptor: [General Practice] explode all trees

#2 ((family or general) NEAR/3 (physician* or doctor* or practitioner* or medicine or practice*))

#3 (GP* or "primary health provider*")

#4 #1 OR #2 OR #3

#5 (nurse-led or nurse-manag* or nurse-delivered or (nurse* NEAR/6 (led or manage* or program* or model* or provision or intervention* or service or system* or care or healthcare or deliver* or treat* or pathway)))

#6 (implementation or evaluation* or barrier* or challenge* or facilitat* or feasib* or enable* or "process evaluation" or "implementation science")

#7 #4 AND #5 AND #6

#8 "sleep health"

#9 MeSH descriptor: [Sleep Apnea, Obstructive] explode all trees

#10 MeSH descriptor: [Sleep Initiation and Maintenance Disorders] explode all trees

#11 MeSH descriptor: [Pulmonary Disease, Chronic Obstructive] explode all trees

#12 (hypertension or "high blood pressure" or "elevated blood pressure" or htn or hypertensive)

#13 MeSH descriptor: [Asthma] explode all trees

#14 MeSH descriptor: [Cardiovascular Diseases] explode all trees

#15 MeSH descriptor: [Neoplasms] explode all trees

#16 (cancer* or carcinoma* or neoplas* or tumo*r* or malignan* or oncolog* or leuk*emia* or metasta* or lymphoma* or melanoma*)

#17 MeSH descriptor: [Diabetes Mellitus] explode all trees

#18 ("heart failure" or copd or coad or "chronic obstructive pulmonary disease" or "chronic obstructive airways disease" or "coronary artery disease" or asthma or "cardiovascular disease" or diabetes or NIDDM or IDDM)

#19 #8 OR #9 OR #10 OR #11 OR #12 OR #13 OR #14 OR #15 OR #16 OR #17 OR #18

#20 #7 AND #19

#21 australia or austria or belgium or bulgaria or canada or croatia or czechia or czech republic or denmark or estonia or finland or france or germany or greece or hungary or iceland or ireland or italy or japan or latvia or lithuania or luxembourg or malta or netherlands or new zealand or norway or poland or portugal or romania or slovakia or slovenia or spain or sweden or switzerland or united kingdom or england or scotland or wales or united states

#22 Australia* or austria* or belgium or belgian or bulgaria* or canad* or croatia* or czechia* or czech republic or denmark or danish or estonia* or finland or finnish or france or french or german or germany or greece or greek or hungar* or iceland* or ireland or irish or italy or italian or japan* or latvia* or lithuania* or luxembourg* or malta or maltese or netherlands or dutch or new zealand or new zealanders or NZ or norway or norwegian or poland or polish or portug* or romania* or slovakia* or slovenia* or spain or spanish or sweden or swedish or switzerland or swiss or united kingdom or UK or england or scotland or scottish or wales or welsh or ireland or irish or united states or USA or US

#23 #21 OR #22

#24 #20 AND #23

*2^nd^ June 2022: records identified: 422*

*4^th^ July 2025: records identified: 92*

Embase

1 general practitioner/ or exp primary health care/ or family medicine/

2 general practice/

3 (((family or general) adj2 (physician* or doctor* or practitioner* or medicine or practice*)) or GP* or "primary health provider*").tw,kf.

4 or/1-3

5 (nurse-led or nurse-manag* or nurse-delivered or (nurse* adj5 (led or manage* or program* or model* or provision or intervention* or service or system* or care or healthcare or deliver* or treat* or pathway))).tw,kf.

6 exp program evaluation/ or implementation science/ or program development/

7 (implementation or evaluation* or barrier* or challenge* or facilitat* or feasib* or enable* or "process evaluation" or "implementation science").tw,kf.

8 or/6-7

9 4 and 5 and 8

10 sleep disordered breathing/

11 ("sleep health" or "obstructive sleep apnea" or "obstructive sleep apnoea").tw,kf.

12 (chronic adj6 insomnia).tw,kf.

13 chronic obstructive lung disease/

14 exp cardiovascular disease/

15 exp hypertension/

16 (hypertension or "high blood pressure" or "elevated blood pressure" or htn).tw,kf.

17 exp asthma/

18 ("heart failure" or copd or coad or "chronic obstructive pulmonary disease" or "chronic obstructive airways disease" or "coronary artery disease" or asthma or "cardiovascular disease").tw,kf.

19 exp diabetes mellitus/

20 (diabetes or NIDDM or IDDM).tw,kf.

21 exp neoplasm/

22 (cancer* or carcinoma* or neoplas* or tumo#r* or malignan* or oncolog* or leuk#emia* or metasta* or lymphoma* or melanoma*).tw,kf.

23 or/10-22

24 9 and 23

25 australia/ or austria/ or belgium/ or bulgaria/ or canada/ or croatia/ or czechia/ or czech republic/ or denmark/ or estonia/ or finland/ or france/ or germany/ or greece/ or hungary/ or iceland/ or ireland/ or italy/ or japan/ or latvia/ or lithuania/ or luxembourg/ or malta/ or netherlands/ or new zealand/ or norway/ or poland/ or portugal/ or romania/ or slovakia/ or slovenia/ or spain/ or sweden/ or switzerland/ or united kingdom/ or england/ or scotland/ or wales/ or united states/

26 24 and 25

*2^nd^ June 2022: records identified: 806*

*4^th^ July 2025: records identified: 132*

ProQuest Theses and Dissertations

noft(( ( ( ( family OR general ) NEAR/2 ( physician* OR doctor* OR practitioner* OR medicine OR practice* ) ) OR gp* OR "primary health provider*" ) AND ( nurse-led OR nurse-manag* OR nurse-delivered OR ( nurs* AND ( "models of care" OR "model of care" OR "care model" ) ) OR ( nurse NEAR/5 ( led OR manage* OR program* OR model* OR provision OR intervention* OR service OR system* OR care OR healthcare OR deliver* OR treat* OR pathway* ) ) ) AND ( "heart failure" OR copd OR coad OR "chronic obstructive pulmonary disease" OR "coronary artery disease" OR "chronic obstructive airways disease" OR asthma OR "cardiovascular disease" OR "congestive heart failure" OR "type 2 diabetes" OR niddm OR diabetes OR iddm OR "type 1 diabetes" OR cancer* OR carcinoma* OR neoplas* OR malignan* OR oncolog* OR leukemia* OR metasta* OR lymphoma* OR melanoma* ) AND ( implementation OR barrier* OR facilitat* OR feasib* OR enable* OR "process evaluation" OR dissemination OR "implementation science" ) AND (Australia* OR Austria* OR Belgium OR Belgian OR Bulgaria* OR Canad* OR Croatia* OR Czechia* OR "Czech Republic" OR Denmark OR Danish OR Estonia* OR Finland OR Finnish OR France OR French OR German* OR Greece OR Greek OR Hungar* OR Iceland* OR Ireland OR Irish OR Japan* OR Italy OR Italian OR Latvia* OR Lithuania* OR Luxembourg* OR Malta OR Maltese OR Netherlands OR Dutch OR "New Zealand*" OR Norway OR Norwegian OR Poland OR Polish OR Portug* OR Romania* OR Slovakia* OR Sloveni* OR Spain OR Spanish OR Sweden OR Swedish OR Switzerland OR Swiss OR "United Kingdom" OR UK OR England OR English OR Scotland OR Scottish OR Wales OR Welsh OR "United States" OR USA) ))

Limit after by publication type -theses

*2^nd^ June 2022: records identified: 33*

*4^th^ July 2025: records identified: 3*
