# Supplementary material for: Factors influencing the implementation of general practice nurse-delivered models of care for chronic conditions: a mixed-methods systematic review to inform models of care for chronic sleep disorders
Source: BMC Prim Care. 2026 Feb 14;27:98. doi: 10.1186/s12875-025-03078-4 (PMC13011425; doi:10.1186/s12875-025-03078-4)
Supplement: Supplementary file 2 — Additional file 2. Data extraction table [file 12875_2025_3078_MOESM2_ESM.docx]

**Data extraction table**

| **First author** | **Year** | **Country of origin** | **Name of chronic condition/s** | **Barriers** | **Facilitators** |
| --- | --- | --- | --- | --- | --- |
| Afzali | 2013 | Australia | Type 2 diabetes |  | Intervention  ***Implementability:*** Practice nurse delivered care is economically viable |
| Alves | 2022 | Portugal | Hypertension | Patient  ***Capability to participate:*** Physical ill health; psychological ill health | Professional  ***Competency:*** Capability to recognise psychological ill health in patients |
| Angus | 2012 | UK | COPD |  | Professional  ***Competency:*** Sufficient training enables confidence in the delivery of the intervention |
| Ansari | 2018 | Australia | COPD | Organisation  ***Resources:*** Limited time to deliver the intervention due to existing busy workloads; computers ill-equipped for use in delivery of the intervention; cost of training staff  ***Involvement:*** Lack of GP support for implementation of the model of care limits the capacity of nurses to be involved  Professional  ***Professional role:*** GP reluctance to delegate responsibility to the nurse due to lack of knowledge about the model of care  ***Nature and characteristics:*** Completion of detailed documentation during appointments is a barrier to delivery of the intervention  Patient  ***Capability to participate:*** Competing health priorities  ***Willingness to participate:*** Unreadiness to make change | Organisation  ***Resources:*** Access to appropriate private space to deliver the intervention; extra administrative assistance; protected time to deliver the intervention  ***Involvement:*** GP involvement in the model of care facilitates a collaborative approach to care delivery; teamwork between nurses and GPs reduces duplication of care delivery  Professional  ***Attitudes to change:*** Belief in the value of the model of care  ***Philosophy of care:*** Motivation to change is a facilitator to engagement of nurses in the model of care  ***Competency:*** Sufficient training enables confidence in the delivery of the intervention; education of the practice team facilitates a team approach to implementation |
| Avery | 2016 | UK | Type 2 diabetes | Organisation  ***Resources:*** Limited time to attend training; lack of time to practice delivering the intervention prior to implementation  Professional  ***Attitudes to change:*** A perception by health care professionals that the intervention only benefits a subgroup of patients  ***Philosophy of care:*** Patient preference not to participate in group treatment  Patient  ***Capability to participate:*** Physical ill health | Organisation  ***Relationship:*** Support and encouragement by health care professionals is a facilitator to patient engagement  ***Involvement:*** Agreement within the practice that the intervention should be delivered by nurses  Professional  ***Professional role:***  ***Competency:*** Sufficient training increases staff awareness of the value of the model of care; completion of training enables skill acquisition; practice delivering the intervention facilitates confidence in intervention delivery  ***Attitudes to change:*** Belief by health care professionals that the intervention will improve patient outcomes  Intervention  ***Nature and characteristics:*** Alignment of the model of care with existing work practices |
| Birgisdóttir | 2025 | Iceland | Type 2 diabetes | External context  ***Fit with local or national agenda:*** Infection risk limited capacity to provide hands on care  Organisation  ***Resources:*** Lack of staff over leave periods halts delivery of care  Patient  ***Capability to participate:*** Lack of access to transport |  |
| Blackberry | 2013 | Australia | Type 2 diabetes | Organisation  ***Resources:*** Limited time capacity despite available funding; lack of appropriate private space to deliver the intervention; implementing new interventions increases workloads  Professional  ***Attitudes to change:*** Competing demands of general practice  Patient  ***Capability to participate:*** Competing health priorities |  |
| Boyle | 2016 | Australia | Type 2 diabetes | Organisation  ***Processes and systems:*** Short appointment times are insufficient for effective delivery of the intervention  Patient  ***Capability to participate:*** Competing health priorities | Organisation  ***Processes and systems:*** Continuity of care is not required for patient engagement if the patient perceives the intervention as task-orientated  Professional  ***Philosophy of care:*** A person-centred approach to care is a facilitator to patient engagement  Intervention  ***Nature and characteristics:*** Structured protocols |
| Carlisle | 2013 | Australia | Type 2 diabetes | External context  ***Policy and legislation:*** Insufficient legislation to enable the development of sustainable services  ***Economic climate and governmental financing:***  Limited funding models; funding model does not suit multidisciplinary models of care | External context  ***Policy and legislation:*** Government recognition of the need for the intervention  ***Infrastructure:*** Adequate IT infrastructure  Organisation  ***Resources:*** Access to existing resources for use in the intervention; ongoing IT support  ***Relationship:*** A prior trusted relationship with the external provider introducing the model of care is a facilitator to practice engagement  Intervention  ***Nature and characteristics:*** Perceived benefits of the model of care for patients facilitates engagement of health care professionals; evidence of benefits to patients; alignment of the model of care with existing work practices  ***Implementability:*** Training to use the technology associated with the intervention  Patient  ***Capability to participate:*** Technology literacy |
| Chimoriya | 2024 | Australia | Type 2 diabetes | External context  ***Fit with local or national agenda:*** Infection risk limited face-to face appointments  Intervention  ***Nature and characteristics:*** Telephone-delivered interventions reduce patient engagement with the intervention  Patient  ***Capability to participate:*** Lack of time to commit to the intervention  ***Willingness to participate:*** Lack of confidence in the feasibility of the intervention limits patient engagement | Organisation  ***Processes and systems:*** Regular appointments provide accountability for patients to adhere to treatment  ***Relationship:*** Support and encouragement by health care professionals is a facilitator to patient engagement; accountability to the health care professional facilitates patient engagement; ongoing support from health professionals facilitates ongoing engagement by patients  ***Skill mix:*** Defined roles for all team members  ***Involvement:*** GP support of the model of care increases patients’ confidence in the intervention  Professional  ***Philosophy of care:*** Active engagement in the model of care by the nurse is a facilitator to patient engagement  ***Competency:*** GPs with sufficient knowledge about the model of care can facilitate patient engagement  Intervention  ***Nature and characteristics:*** Evidence of benefits to patients; models of care that produce quick results are motivating for patients; models of care with tangible results facilitate patient engagement; face-to-face delivery of an intervention facilitates motivation in patients; subsidised treatment is a facilitator to patient engagement  Patient  ***Capability to participate:*** Support from family facilitates adherence to treatment  ***Willingness to participate:*** To improve their health status; to reduce their use of medication |
| Chmiel | 2017 | Switzerland | Type 2 diabetes | External context  ***Economic climate and governmental financing:*** Lack of funding for nurse-delivered care  Organisation  ***Resources:*** Lack of appropriate private space to deliver the intervention; cost of set up for the model of care without established funding  ***Skill mix:*** Staffing changes during the delivery of the intervention  Professional  ***Professional role:*** GP reluctance to delegate responsibility to the nurse due to a fear of loss of control; GP reluctance to delegate responsibility to the nurse due to insufficient workload of their own; a shift from a hierarchical relationship to a model with a nurse at the centre of care can be challenging for GPs  ***Philosophy of care:*** Patient preference to only see GP for care  Intervention  ***Nature and characteristics:*** Lack of integration into practice electronic medical record software can result in reduced use by clinicians; the intervention is complicated to deliver  Patient  ***Willingness to participate:*** Unwillingness to engage with the model of care | Professional  ***Competency:*** Training of the practice team facilitates a team approach to implementation |
| Christianson | 1997 | USA | Hypertension | Organisation  ***Involvement:*** Not having clinicians in planning meetings for the implementation of the intervention delays important changes to protocols  Professional  ***Professional role:*** Lack of definition of the scope of practice of the enhanced role of the nurse  ***Attitudes to change:*** Health care professional lack of confidence about the effectiveness of the intervention; Perception by GPs that the intervention lacks benefit  Intervention  ***Implementability:*** Lack of funding to commence model of care | Organisation  ***Culture:*** Regular team meetings  ***Involvement:*** Engagement of individuals with decision making power in implementation meetings  ***Processes and systems:*** Consistent, efficient and transparent billing systems reduces clinician workloads; scheduling of patient appointments by nurses could enhance the nurse-patient relationship  Intervention  ***Nature and characteristics:*** Perceived benefits of the model of care for patients facilitates engagement of health care professionals; the provision of resources to patients at no cost is a facilitator for GP engagement |
| Ciccone | 2010 | Italy | Cardiovascular disease, diabetes, heart failure |  | Organisation  ***Relationship:*** A strong nurse-patient relationship is a facilitator to effective delivery of the model of care; collaborative nurse-doctor relationship  Professional  ***Philosophy of care:*** An empathetic approach to care facilitates patient engagement with a model of care  Intervention  ***Nature and characteristics:*** Evidence of benefits to patients |
| Clarke | 2019 | UK | Prostate cancer | Organisation  ***Resources:*** Cost of training staff; slow internet access  ***Relationship:*** Poor communication between general practice and specialist services is a barrier to accessing support from specialist services; concern that delivery of the intervention could damage the health care professional-patient relationship  Professional  ***Attitudes to change:*** Health care professional uncertainty about patient capacity to engage with an online intervention  ***Competency:*** Lack of knowledge limits capacity to deliver the intervention  Patient  ***Capability to participate:*** Language barrier; visual impairment; pain that impairs capacity to engage with the intervention; technology illiteracy; lack of access to the technology needed for the intervention  ***Relevance to self:*** A belief that their condition is mild and doesn’t require treatment; a belief that their condition is too longstanding to be suitable for the intervention  ***Willingness to participate:*** Avoidance of the intervention due to it being an unwelcome reminder of their health issues | Organisation  ***Resources:*** Ongoing IT support  ***Relationship:*** Support from specialist staff improves practice staff confidence  Professional  ***Competency:*** Sufficient training enables confidence in the delivery of the intervention  Intervention  ***Nature and characteristics:*** Perceived benefits of the model of care for patients facilitates engagement of health care professionals; a model of care that facilitates shared care with specialist services and the patient is a facilitator for nurse engagement; capability to complete documentation online prior to appointments is a facilitator for patient engagement; face-to-face delivery of an intervention facilitates the nurse-patient relationship; linkage with practice electronic medical records software  Patient  ***Willingness to participate:*** A belief that the model of care frees up health professional time |
| Clarke | 2020 | UK | Prostate cancer | Organisation  ***Skill mix:*** Delivery of the intervention by nurses employed outside of the general practice limits the development of the nurse-patient relationship  Intervention  ***Nature and characteristics:*** Intervention software that is not user-friendly; lack of integration into practice electronic medical record software can result in reduced use by clinicians  ***Implementability:*** Offering limited dates for training can result in not all staff being able to be trained to implement the model of care | Intervention  ***Nature and characteristics:*** Easy to use resources; telephone-delivered interventions are convenient for patients |
| Crowley | 2012 | United States | Type 2 diabetes | Organisation  ***Skill mix:*** Delivery of care by nurses working remotely and not employed by the practice limits relationships with GPs |  |
| Dellasega | 2010 | USA | Type 2 diabetes | Patient  ***Willingness to participate:*** Unreadiness to make change | Organisation  ***Processes and systems:*** Longer appointment times facilitate the development of the nurse-patient relationship  Professional  ***Philosophy of care:*** A calm approach to care delivery facilitates ongoing patient engagement; a person-centred approach to care is a facilitator to patient engagement; an empathetic approach to care facilitates patient engagement with a model of care |
| Dontje | 2013 | United States | Coronary artery disease | Organisation  ***Resources:*** Implementing new interventions increases workloads  Intervention  ***Nature and characteristics:*** Implementing new interventions increases workloads; costs associated with an intervention are a barrier to patient engagement | Organisation  ***Processes and systems:*** Alignment of nurse availability with patient availability |
| Eley | 2013 | Australia | Type 2 diabetes, HT and ischaemic heart disease | Intervention  ***Implementability:*** Extra time needed to embed the model of care into routine practice | Organisation  ***Processes and systems:*** Longer appointment times facilitate patient engagement with models of care  ***Skill mix:*** Shared responsibilities between team members  ***Involvement:*** GP support of the model of care increases patients’ confidence in the intervention; Evidence of teamwork between GPs and nurses instils confidence in patients  Patient  ***Willingness to participate:*** A belief that the model of care frees up health professional time |
| Faulkner | 2016 | UK | COPD | Organisation  ***Resources:*** A lack of clinical time reduces nurse confidence in the delivery of the intervention; access to poor quality equipment only; limited time to deliver the intervention due to existing busy workloads; limited time to attend training  ***Skill mix:*** Working alone on the model of care limits a nurse’s capacity to receive clinical support from colleagues  ***Involvement:*** Lack of support from nursing colleagues is a barrier to nurse confidence in the delivery of the intervention; lack of support from the GP is a barrier to nurse confidence in the delivery of the intervention; lack of autonomy by nurses to be flexible with appointment length; lack of allocated time with the GP to discuss patient progress  Professional  ***Competency:*** Insufficient training leads to lack of confidence in the delivery of the intervention; lack of knowledge limits the ability of GPs to support the nurse to deliver the intervention  Intervention  ***Nature and characteristics:*** The intervention is time consuming  ***Implementability:*** Offering limited education within work hours | External context  ***Infrastructure:*** Larger-sized practices are associated with greater confidence in nurses  Organisation  ***Resources:*** Sufficient time for interpretation of results improves nurse confidence in the delivery of the intervention  ***Skill mix:*** Nurses with less than eleven years of experience have greater confidence in delivery of an intervention  ***Involvement:*** Support from GPs improves nurse confidence in the delivery of the intervention; nursing team support improves nurse confidence; capacity for nurses to amend appointment length as needed  Professional  ***Competency:*** The receipt of external training is a facilitator to nurse confidence in the delivery of the intervention; ongoing training facilitates confidence in the delivery of interventions |
| Furler | 2014 | Australia | Type 2 diabetes | Intervention  ***Implementability:*** Implementing a new model of care is time consuming  Patient  ***Capability to participate:*** Competing health priorities; competing caring responsibilities | External context  ***Economic climate and governmental financing:*** Funding of interventions to fund the salaries of practice nurses  Organisation  ***Processes and systems:*** Continuity of care is a facilitator to success of the model of care; continuity in the working relationship between nurses and GPs facilitates teamwork  ***Skill mix:*** Agreement about the accountability of the intervention  ***Relationship:*** A successful nurse-patient relationship improves patient engagement with the model of care; trust is a facilitator to patient engagement; trust between nurse and GP facilitates teamwork; familiarity with the nurse is a facilitator to patient engagement  Intervention  ***Nature and characteristics:*** Introduction of the intervention over several appointments reduces the risk of overwhelming patients; patient resources that can be adapted to meet individual patient needs; opportunity for patients to connect with other patients receiving the intervention |
| Gianfrancesco | 2020 | UK | Type 2 diabetes | Organisation  ***Resources:*** Limited time to deliver the intervention due to existing busy workloads; lack of time limits nurse capacity to seek out support from specialist staff  ***Relationship:*** Minimal communication with specialist care results in a lack of clarity about the role of the specialist provider in care delivery; a limited relationship with specialist providers limits nurse capacity to determine their competence in the delivery of the intervention  ***Involvement:*** Lack of support from team members is associated with time pressure in nursing appointments; lack of support from the GP is a barrier to nurse confidence in the delivery of the intervention  Professional  ***Competency:*** Lack of skills in nurses is associated with time pressure in appointments; insufficient knowledge leads to a lack of confidence in the delivery of the intervention by nurses | Organisation  ***Relationship:*** Support from specialist staff improves practice staff confidence  ***Involvement:*** Support from GPs improves nurse confidence in the delivery of the intervention  Professional  ***Attitudes to change:*** Receipt of positive patient feedback is facilitator to nurse confidence in their delivery of the intervention  Intervention  ***Nature and characteristics:*** Perceived benefits of the model of care for patients facilitates engagement of health care professionals |
| Gilles de La Londe | 2023 | France | Type 2 diabetes |  | Organisation  ***Involvement:*** Collaboration between GPs and nurses improves the quality of the care that they deliver |
| Goff | 2020 | UK | Type 2 diabetes | External context  ***Financial incentives:*** Practice incentives that offer financial incentive but do not address the needs of patients  Organisation  ***Resources:*** Limited time to deliver the intervention due to existing busy workloads; limited time to attend training  ***Processes and systems:*** Short appointments are a barrier to patient engagement; short appointment times limit the development of trust between the health care professional and the patient  ***Culture:*** General practice is an environment of constant change  Professional  ***Philosophy of care:*** Lack of accountability for self-management by patients makes delivery of the intervention difficult; a conflict between patient’s belief system and the intervention limits patient’s engagement with the model of care; a conflict between the patient’s cultural identity and the intervention  Intervention  ***Nature and characteristics:*** Use of unfamiliar terminology is a barrier for some patients  Patient  ***Capability to participate:*** Competing caring responsibilities  ***Willingness to participate:*** Fear of the intervention; fear of social stigma | Organisation  ***Skill mix:*** The delivery of a culturally-specific intervention by nurses with that cultural identity may facilitate the development of the nurse-patient relationship  Professional  ***Competency:*** Formal cultural skills training is needed to successfully deliver culture-specific care  Intervention  ***Nature and characteristics:*** Patient resources that can be adapted to meet individual patient needs |
| Graves | 2016 | UK | Type 2 diabetes | Organisation  ***Resources:*** Limited time to deliver the intervention due to existing busy workloads  ***Involvement:*** Lack of support from the GP reduces nurse interest in being involved in the model of care  Professional  ***Professional role:*** Concern by nurses about working outside of their scope of practice; perception by nurses that GPs do not value their role within the model of care  ***Attitudes to change:*** Fear by nurses that delivering the intervention could be perceived as if they are not prioritising their regular work  Intervention  ***Implementability:*** Implementing a new model of care adds work to existing workload  Patient  ***Capability to participate:*** Low intelligence  ***Relevance to self:*** The intervention is not a priority in the context of other commitments unreadiness to make change  ***Willingness to participate:*** Non-attendance at appointments | Organisation  ***Processes and systems:*** Longer appointment times allow for more comprehensive delivery of the intervention; longer appointment times enable the delivery of patient-centred care; extra administrative assistance  ***Relationship:*** Support from specialist staff improves practice staff confidence  ***Involvement:*** Effective teamwork within the practice |
| Greaves | 2003 | UK | Type 2 diabetes | Organisation  ***Resources:*** Limited time to deliver the intervention due to existing busy workloads; busy workloads limit GP capacity to support nurses to deliver the intervention  ***Skill mix:*** Part time staffing limits capacity to provide prompt patient follow up; working alone on the model of care limits a nurse’s ability to ascertain their competence in the delivery of the intervention; insufficient opportunities to deliver the intervention can result in difficulty maintaining competency  Professional  ***Competency:*** Lack of knowledge limits the ability of GPs to support the nurse to deliver the intervention  ***Professional role:*** Concerns by nurses about impact on professional indemnity insurance if their scope of practice is not clearly defined | External context  ***Financial incentives:*** Increased financial renumeration for nurses to reflect increased responsibility associated with new models of care  Organisation  ***Resources:*** Protected time to deliver the intervention  ***Processes and systems:*** Continuity of care is a facilitator for nurse engagement  ***Involvement:*** Effective teamwork within the practice; support from GPs improves nurse confidence in the delivery of the intervention  ***Relationship:*** Support from specialist staff improves practice staff confidence  Professional  ***Competency:*** Sufficient training enables nurses to be confident in accepting accountability for care; Ongoing training facilitates confidence in the delivery of interventions; Formal training with supplemental practical supervised training facilitates competence |
| Halcomb | 2007 | Australia | Cardiovascular disease | External context  ***Infrastructure:*** Lack of standardised databases and software in general practice  ***Economic climate and governmental financing:*** Limited funding models  Organisation  ***Involvement:*** Lack of teamwork | External context  ***Infrastructure:*** Consistent funding for practice nurse activities within general practice  ***Financial incentives:*** Standardised renumeration for practice nurses; renumeration for nurses that includes support to undertake professional development  Organisation  ***Skill mix:*** Defined roles for all team members  Professional  ***Professional role:*** GP awareness of the scope of practice specific to nurse classifications; presence of competency statements that define the scope of practice of the nurse  Intervention  ***Nature and characteristics:*** Structured protocols  ***Implementability:*** Training that is offered with multiple modes of delivery |
| Halcomb | 2008 | Australia | Cardiovascular disease | External context  ***Economic climate and governmental financing:*** Lack of funding for nurse-delivered care  ***Financial incentives:*** Inadequate financial renumeration for nurses to reflect the increased responsibility associated with new models of care  Organisation  ***Resources:*** Limited time to deliver the intervention due to existing busy workloads; lack of appropriate private space to deliver the intervention; busy workloads make finding time to establish the model of care challenging  ***Skill mix:*** A lack of definition of team members’ roles can contribute to poor collaboration between nurses and GPs  Professional  ***Professional role:*** GP reluctance to delegate responsibility to the nurse due to lack of knowledge about the nursing scope of practice; perception by nurses that GPs do not value their role within the model of care  ***Attitudes to change:*** Lack of interest by nurses in extending their scope of practice to deliver new models of care; A lack of interest by GPs in being involved in new models of care due to impact on existing work practices | Organisation  ***Involvement:*** Support from GPs improves nurse confidence in the delivery of the intervention  Professional  ***Professional role:*** A commitment to their role is a facilitator to nurse engagement  ***Attitudes to change:*** Receipt of patient feedback is a motivator for nurse engagement  ***Competency:*** Sufficient training enables confidence in the delivery of the intervention |
| Hardeman | 2014 | UK | Type 2 diabetes | Intervention  ***Nature and characteristics:*** Scripted interventions are a barrier to person-centred care |  |
| Hegney | 2013 | Australia | type 2 diabetes, stable IHD or hypertension | External context  ***Economic climate and governmental financing:*** Lack of funding for nurse-delivered care | Organisation  ***Resources:*** Access to appropriate private space to deliver the intervention  ***Processes and systems:*** Longer appointment times enable the delivery of patient-centred care; continuity of care is a facilitator to patient engagement  ***Relationship:*** Respect and trust between team members; GP confidence in the nurse facilitates patient confidence in the nurse; effective communication by the nurse is a facilitator to patient confidence in the competency of the nurse  ***Skill mix:*** Employment of staff who display teamwork skills; shared responsibilities between team members  ***Involvement:*** GP involvement in the model of care increases patients’ confidence in the intervention  Professional  ***Professional role:*** Nurses working within their scope of practice facilitates GP support for nurse-delivered models of care  ***Attitudes to change:*** An interest in the intervention  ***Competency:*** Sufficient training is a facilitator to patient confidence in the nurse  ***Philosophy of care:*** Perception of teamwork with the nurse improves engagement with the model of care by patients; ; a person-centred approach to care is a facilitator to patient engagement  Intervention  ***Nature and characteristics***: A model of care that reduces GP workload is a facilitator to staff engagement; regular appointments enable greater patient accountability for their role within the model of care; structured protocols  ***Implementability:*** An initial investment of time allows for the nurse to develop confidence in the delivery of the intervention  Patient  ***Relevance to self:*** Patients that perceive themselves as having a stable condition  ***Willingness to participate:*** A belief that the model of care frees up health professional time |
| Helmink | 2012 | The Netherlands | Type 2 diabetes |  | External context  ***Stakeholder buy-in:*** Less institutional support is associated with greater motivation by practices to implement and maintain an intervention  Organisation  ***Processes and systems: C***ompatibility of the intervention with existing work practices  ***Involvement:*** Support from colleagues increases staff motivation to deliver the intervention  Professional  ***Attitudes to change:*** Females are more motivated to implement a model of care than men; greater self-efficacy prior to implementation is related to motivation to continue to deliver the intervention  Intervention  ***Nature and characteristics:*** Perceived benefits of the model of care to general practice staff |
| Hogervorst | 2021 | The Netherlands | Type 2 diabetes | Organisation  ***Resources:*** Limited time to deliver the intervention due to existing busy workloads; lack of capacity within electronic medical record systems to screen for suitable patients  ***Relationship:*** Lack of established relationships with key local stakeholders  ***Involvement:*** Lack of GP support for implementation of the model of care limits the capacity of nurses to be involved  Professional  ***Attitudes to change:*** Time invested in non-adherent patients is not seen as valuable for the clinical outcomes achieved; Time invested in the implementation for minimal patients reduces GP engagement with the intervention; uncertainty about the cost-effectiveness of the model of care  ***Competence:*** Lack of knowledge about how to address non-adherence to the intervention  Intervention  ***Nature and characteristics:*** Manual screening for suitable patients is laborious  Patient  ***Capability to participate:*** Multiple health issues; addiction; language barrier | Organisation  ***Involvement:*** GP support of the model of care enables nurses to dedicate more time to the delivery of the intervention  Professional  ***Philosophy of care:*** Patient perception of nurse competence is a facilitator to a trusting nurse-patient relationship; active engagement in the model of care by the nurse is a facilitator to a trusting nurse-patient relationship  Intervention  ***Nature and characteristics:*** Structured protocols; the provision of guidelines for management of non-adherence to the intervention |
| Hoskins | 2016 | UK | Asthma | Organisation  ***Resources:*** Implementing new interventions increases workloads; complex processes associated with models of care add demand to an already busy practice  ***Processes and systems:*** Short appointment times are insufficient for effective delivery of the intervention  Intervention  ***Nature and characteristics:*** Use of unfamiliar terminology is a barrier for some patients; evaluating patient information and developing patient-specific plans is difficult to achieve in one appointment  Patient  ***Capability to participate:*** Competing health priorities  ***Relevance to self:*** A belief that their condition is mild and doesn’t require treatment; a belief that they are too old for the intervention | Professional  ***Philosophy of care:*** A person-centred approach to care is a facilitator to patient engagement; A person-centred approach to care facilitates the development of the nurse-patient relationship  Intervention  ***Nature and characteristics:*** Alignment of the model of care with existing work practices |
| Ismail | 2018 | UK | Type 2 diabetes | External context  ***Fit with local or national agenda:*** Implementing a model of care during restructuring of primary care services reduces engagement by practices due to uncertainty  Organisation  ***Resources:*** Limited time capacity despite available funding  ***Relationship:*** An ineffective nurse-patient relationship is a barrier to patient engagement  Professional  ***Professional role:*** Concern by nurses about working outside of their scope of practice  ***Attitudes to change:*** Resentment about the additional workload associated with the model of care  ***Competency:*** Insufficient training leads to lack of confidence in the delivery of the intervention  Patient  ***Willingness to participate:*** Lack of engagement with the model of care |  |
| Jansink | 2010 | The Netherlands | Type 2 diabetes | Organisation  ***Resources:*** Busy workloads limit nurse capacity to provide patient centred care; limited time to attend training; loss of income related to staff attending training  ***Skill mix:*** Lack of clarity about team members roles  ***Relationship:*** Concern that delivery of the intervention could damage the health care professional-patient relationship  Professional  ***Competency:*** Lack of knowledge limits capacity to deliver the intervention; lack of empathy due to lack of personal experience  Intervention  ***Nature and characteristics:*** Inadequate guidelines for effective delivery of the intervention; lack of immediate results limits patient engagement  Patient  ***Capability to participate:*** Language barrier; low literacy; low socio-economic status; lack of access to the resources needed to participate in the intervention; addiction; lack of discipline, presence of stress  ***Willingness to participate:*** Peer pressure by others not to participate; **l**ack of engagement by patients leads to lack of motivation by staff |  |
| Jansink | 2013 | The Netherlands | Type 2 diabetes |  | Intervention  ***Nature and characteristics:*** Structured protocols |
| Jones | 1995 | UK | Asthma | Organisation  ***Resources:*** Limited time to deliver the intervention due to existing busy workloads; Lack of appropriate private space to deliver the intervention  ***Involvement:*** Lack of involvement of administrative staff in the model of care  Professional  ***Professional role:*** Lack of definition of the scope of practice of the enhanced role of the nurse  Intervention  ***Implementability:*** Implementing a new model of care is time consuming | Organisation  ***Resources:*** Extra administrative assistance |
| Katon | 2010 | USA | Diabetes, coronary heart disease |  | Organisation  ***Culture:*** Regular team meetings |
| Kendall | 2010 | UK | Diabetes, asthma, COPD, heart disease | Organisation  ***Processes and systems:*** Inflexibility in nursing appointment schedules limits patient access to care  ***Skill mix:*** Part time staffing limits capacity to provide prompt patient follow up  Patient  ***Capability to participate:*** Receipt of interventions for other health conditions with advice that conflicts with the model of care |  |
| Kenealy | 2004 | New Zealand | Diabetes | Patient  ***Willingness to participate:*** Non-adherence to treatment; lack of engagement with the model of care |  |
| Kolltveit | 2023 | Norway | COPD | External context  ***Economic climate and governmental financing:*** Lack of funding for team-based activities delivered by nurses  Organisation  ***Resources:*** Limited time to deliver the intervention due to existing busy workloads  ***Involvement:*** Working collaboratively with nurses increases GP workloads  Professional  ***Competency:*** Time consuming training; insufficient training leads to lack of confidence in the delivery of the intervention | External context  **Economic climate and governmental financing:** Funding for nurse-delivered interventions  Professional  ***Involvement:*** Effective teamwork within the practice  ***Philosophy of care:*** Active engagement in the model of care by the nurse is a facilitator to patient engagement; a person-centred approach to care is facilitator to patient engagement; shared decision making with the patient is a facilitator to patient engagement  Intervention  ***Nature and characteristics:*** Use of structured protocols is a facilitator to increased knowledge and skills of nurses |
| Kolltveit | 2024 | Norway | Type 2 diabetes | External context  ***Fit with local or national agenda:*** COVID-19 increased workloads of general practice staff  Organisation  ***Resources:*** Limited time to deliver the intervention due to existing busy workloads  ***Processes and systems:*** Lack of communication about appointment scheduling for GPs and nurses increases work pressure for GPs; long appointments are expensive to deliver; misalignment of GP and nurse schedules is a barrier to collaborative practice  ***Skill mix:*** A lack of definition of team members’ roles can contribute to poor collaboration between nurses and GPs | External context  ***Economic climate and governmental financing:*** Adequate funding is a facilitator to delivering long appointments  Organisation  ***Processes and systems:*** Longer appointment times enable the delivery of patient-centred care  ***Relationships:*** Effective communication between GPs and nurses is a facilitator to a collaborative working relationship; face-to-face communication between GPs and nurses is a facilitator to a collaborative working relationship  ***Skill mix:*** Defining the responsibilities of team members is a facilitator to collaborative care  ***Involvement:*** Working collaboratively increases the skills and knowledge of GPs and nurses; collaboration between GPs and nurses improves the quality of the care that they deliver  Professional  ***Attitudes to change:*** Belief by health care professionals that the intervention will improve patient outcomes  ***Competency:*** Confidence in a nurse’s competence is a facilitator to GP engagement; Opportunities to exchange skills and knowledge between team members is a facilitator to staff engagement  Intervention  ***Nature and characteristics:*** A shared medical record facilitates collaboration between nurses and GPs; use of a standardised approach to documentation facilitates delivery of care by GPs and nurses; a model of care that enables sharing skills and knowledge between health care professionals is a facilitator to staff engagement |
| Kyle | 2024 | UK | Insomnia | External context  ***Fit with local or national agenda:*** Lockdowns due to COVID-19 made delivery of an intervention challenging  Organisation  ***Resources:*** Limited time to deliver the intervention due to existing busy workloads  ***Processes and systems:*** Difficulty scheduling long appointments into existing short consultation times  Intervention  ***Nature and characteristics:*** The intervention is too demanding for patients  Implementability: A delay between training and implementation  Patient  ***Capability to participate:*** Competing health priorities; lack of self-efficacy  ***Willingness to participate:*** Not prioritising the intervention | Organisation  ***Processes and systems:*** Flexibility in appointment scheduling  ***Relationship:*** Ongoing support from health professionals facilitates adherence to treatment  ***Involvement:*** Nursing team support improves nurse confidence  Professional  ***Competence:*** Sufficient training enables confidence in the delivery of the intervention  Intervention  ***Nature and characteristics:*** Face-to-face delivery of an intervention facilitates motivation in patients; models of care that can be adapted to meet individual patient needs facilitate patient engagement; a model of care that reduces GP workload is a facilitator to staff engagement; scheduling appointments on specific times and days improves practice organisation  Patient  Capability to engage: Self-motivation |
| Lofdahl | 2010 | Sweden | Asthma |  | Organisation  ***Skill mix:*** The delivery of an intervention by a trained specialist nurse enables early initiation of treatment and patient support |
| Molina-Vázquez | 2025 | Spain | COPD | External context  ***Infrastructure***  Requiring institutional approval is a barrier to making change to electronic medical software  Organisation  ***Relationship:*** Limited communication from hospitals; poor relationship between the general practice team and specialist teams  ***Skill mix:*** Staffing changes during the delivery of the intervention  Professional  ***Competency:*** Lack of knowledge about relevant guidelines; lack of knowledge about best practice care  Intervention  ***Nature and characteristics:*** Ineffective electronic medical software; lack of structured documentation  ***Implementability:*** Commencement of implementation during holiday periods | Professional  ***Competency:*** Providing professional development credit is a facilitator to clinician engagement in training |
| Morrow | 2017 | UK | Asthma | Organisation  ***Resources:*** Limited time to deliver the intervention due to existing busy workloads; lack of access to the equipment needed to deliver the intervention; difficulties finding staff to cover for nurses to attend training; limited time to provide care for patients with complex health needs  ***Skill mix:*** Delegation of responsibilities to the nurse can result in a loss of GP knowledge  Professional  ***Attitudes to change:*** Competing demands of general practice  Intervention  ***Nature and characteristics:*** Patient resources that cannot be adapted to meet individual patient needs have reduced use by patients; poor integration of documentation with practice electronic medical software makes the delivery of the intervention more time consuming; alert fatigue from software reminders; inadequate patient resources for patients with a different first language; duplication of documentation; documentation that does not align with clinical care  Patient  ***Relevance to self:*** A belief that their condition is not serious enough to need the intervention  ***Willingness to participate:*** Lack of engagement with the model of care | Professional  ***Competency:*** Training of the practice team facilitates a team approach to implementation  ***Philosophy of care:*** A person-centred approach to care is a facilitator to patient engagement  Intervention  ***Nature and characteristics:*** Easy to use resources; alignment of the model of care with existing work practices; linkage with practice electronic medical records software |
| Murchie | 2005 | UK | Coronary heart disease | External context  ***Infrastructure:*** Lack of governmental support to enable ongoing delivery of the model of care  Organisation  ***Resources:*** Limited time to attend training; busy workloads make finding time to establish the model of care challenging; nursing staff shortages; lack of appropriate private space to deliver the intervention; insufficient administrative support  ***Culture:*** Changing priorities within the practice  ***Skill mix:*** Insufficient trained staff results in cessation of the intervention in the case of staffing changes; working alone on the model of care limits a nurse’s ability to ascertain their competence in the delivery of the intervention  ***Involvement:*** Lack of support from the GP leads to feelings of isolation in nurses  Professional  ***Professional role:*** GP reluctance to delegate responsibility to the nurse due to desire to provide chronic disease management themselves  ***Attitudes to change:*** Lack of interest in new models of care by GPs  ***Competency:*** Insufficient training limits capacity to deliver the intervention  Intervention  ***Nature and characteristics:*** Lack of financial benefit for GPs | Organisation  ***Processes and systems:*** Continuity of care is a facilitator for nurse engagement  ***Involvement:*** A willingness by GPs to delegate chronic disease management to nurses is needed to sustain a model of care ongoing; GP support of the model of care facilitates delivery of the intervention by the nurse  Professional  ***Attitudes to change:*** Having an opportunity to develop new skills is a motivator for nurses  Intervention  ***Nature and characteristics:*** Evidence of benefits to patients; perceived benefits of the model of care for patients facilitates engagement of health care professionals |
| Nissen | 2024 | Denmark | Type 2 diabetes | Professional  ***Competency:*** Lack of GP knowledge about an intervention is a barrier to GP delivery of an intervention  Intervention  ***Implementability:*** Extra time needed to embed the model of care into routine practice  Patient  ***Capability to participate:*** Technology illiteracy; lack of access to the technology needed for the intervention | Professional  ***Competency:*** Nurses with adequate knowledge and skills require less guidance from GPs  Intervention  ***Nature and characteristics:*** Structured protocols; use of structured protocols is a facilitator to patient confidence in nurse delivery of care; use of structured protocols is a facilitator to nurse confidence in the delivery of an intervention; use of structured protocols can reduce the frequency of appointments; use of structured protocols is a facilitator to nurse autonomy |
| Nurmeksela | 2021 | Finland | Coronary artery disease | Professional  ***Philosophy of care:*** Lack of a person-centred approach to care reduces patient satisfaction  Patient  ***Capability to participate:*** Receipt of interventions for other health conditions with advice that conflicts with the model of care |  |
| Odnoletkova | 2016 | Belgium | Type 2 diabetes | External context  ***Policy and legislation:*** Lack of legal framework to support delivery of the intervention  ***Economic climate and governmental financing:*** Funding model does not suit multidisciplinary models of care  Organisation  ***Resources:*** Busy workloads make finding time to establish the model of care challenging  ***Involvement:*** Lack of GP cooperation in the model of care increases nurse time in the delivery of the intervention  Professional  ***Professional role:*** Uncertainly by nurses about providing recommendations for care to GPs  ***Attitudes to change:*** Competing demands of general practice  Intervention  ***Nature and characteristics:*** Paper documentation is a barrier to integration of documents into practice electronic medical record software; telephone-delivered interventions are not a substitute for face-to-face delivered care; telephone-delivered interventions limit the development of a trusting nurse-patient relationship; telephone-delivered interventions reduce patient engagement with the intervention; the intervention is time consuming  ***Implementability:*** Implementing a new model of care is time consuming  Patient  ***Willingness to participate:*** Non-attendance at appointments; lack of motivation | Intervention  ***Nature and characteristics:*** Telephone-delivered interventions are convenient for patients; a model of care that does not require a significant investment of time is a facilitator for GP engagement; access to data related to patient progress provides motivation to nurses |
| O'Hare | 2004 | UK | Type 2 diabetes |  | Organisation  ***Resources:*** Protected time to deliver the intervention  ***Involvement:*** Involvement of a cultural link worker can facilitate patient adherence in culture-specific patient groups; involvement of a specialist nurse can facilitate patient engagement  Professional  ***Competency:*** Sufficient training can be a facilitator to patient engagement  Intervention  ***Nature and characteristics:*** Structured protocols |
| Oyegbami | 2019 | United States | Type 2 diabetes | Patient  ***Capability to participate:*** Lack of time to commit to the intervention; lack of access to the resources needed to participate in the intervention | Organisation  ***Resources:*** Financial support for patients to access transport for appointments  ***Processes and systems:*** Booking appointments ahead of time to improve patient attendance; conducting phone reminders to improve patient attendance at appointments  Patient  ***Relevance to self:*** A belief by patients that the intervention is useful |
| Perry | 2008 | UK | Cancer | External context  ***Fit with local or national agenda:*** Introduction of the model of care at a time of concern about the nursing profession  Organisation  ***Skill mix:*** Lack of consultation about the scope of the new nurse role can lead to concern about its impact on the role of other team members; lack of a formal introduction of the nursing role results in a lack of clarity about how the role fits into the broader team  Professional  ***Professional role:*** Lack of definition of the scope of practice of the enhanced role of the nurse  ***Competency:*** Lack of knowledge about external organisations is a barrier to development of effective relationships with those organisations |  |
| Persell | 2023 | US | Hypertension | Organisation  ***Resources:*** Limited time to deliver the intervention due to existing busy workloads  Professional  ***Competency:*** Lack of GP knowledge about the referral process; Insufficient training limits capacity to deliver the intervention  Intervention  ***Nature and characteristics:*** Uncertainty about the cost of the intervention for patients limits staff engagement; the intervention is time consuming; the intervention is too demanding for patients  Patient  ***Capability to participate:*** Visual impairment  ***Willingness to participate:*** Lack of engagement with the model of care | Organisation  ***Resources:*** A dedicated staff member to coordinate intervention delivery  ***Processes and systems:*** Availability of appointments with nurses  Involvement; GP involvement in the model of care increases patients’ confidence in the intervention  Patient  ***Relevance to self:*** A belief by patients that the intervention is needed |
| Pinnock | 2005 | UK | Asthma | Organisation  ***Resources:*** Lack of appropriate private space to deliver the intervention | Intervention  ***Nature and characteristics:*** Telephone-delivered interventions work for busy patients; telephone-delivered interventions work for stable patients; face-to-face delivery of an intervention is suitable for patients with unstable conditions; face-to-face delivery of an intervention facilitates the nurse-patient relationship |
| Poskiparta | 2006 | Finland | Type 2 diabetes | Professional  ***Attitudes to change:*** Lack of engagement by nurses and GPs  ***Competency:*** Limited health professional communication skills  Patient  ***Capability to participate:*** Limited conversational skills |  |
| Ratanawongsa | 2012 | United States | Type 2 diabetes | Organisation  ***Resources:*** Limited time to deliver the intervention due to existing busy workloads  ***Involvement:*** Lack of teamwork  Professional  ***Attitudes to change:*** Competing demands of general practice  ***Competency:*** Lack of knowledge limits capacity to deliver the intervention; lack of knowledge about the influences of culture on a patient’s approach to chronic disease management  Intervention  ***Nature and characteristics:*** Inadequate patient resources for patients with limited literacy; inadequate patient resources for patients with a different first language; infrequent appointments limit monitoring of patient progress  Patient  ***Capability to participate:*** Limited health literacy; language barrier  ***Willingness to participate:*** Non-attendance at appointments | Organisation  ***Resources:*** Protected time to deliver the intervention |
| Rehackova | 2022 | UK | Type 2 diabetes | Professional  ***Attitudes to change:*** Health care professional lack of confidence about the effectiveness of the intervention; concern about the safety of the intervention is a barrier to staff engagement; uncertainty of the model of care by peers limits engagement by staff  Intervention  ***Implementability:*** Intensive training can be overwhelming for practice staff | Organisation  ***Relationship:*** A prior trusted relationship with the external provider introducing the model of care is a facilitator to practice engagement; support from specialist staff improves practice staff confidence; support from specialist staff improves practice workload  Professional  ***Competency:*** Formal training with supplemental practical on the job training improves engagement by general practice staff  Intervention  ***Nature and characteristics:*** A demonstrated evidence base for the model of care improves practice engagement with implementation  ***Implementability:*** The provision of initial training and guidance enables the implementation of models of care in practices without existing infrastructure |
| Rehackova | 2022a | United Kingdom | Type 2 diabetes | Intervention  ***Nature and characteristics:*** An intervention that is delivered over a long time is a barrier to patient engagement; lack of sustainability is a barrier to patient engagement | Organisation  ***Relationship:*** Trust is a facilitator to patient engagement; support and encouragement by health care professionals is a facilitator to patient engagement; accountability to the health care professional facilitates patient engagement  ***Processes and systems:*** Regular appointments facilitate patient engagement; continuity of care facilitates trust in the nurse-patient relationship; continuity of care is a facilitator to rapport with patients; continuity of care is a facilitator to patient-centred care  Professional  ***Philosophy of care:*** A person-centred approach to care is facilitator to patient engagement  Intervention  ***Nature and characteristics:*** Demonstration of evidence about the intervention facilitates patient engagement; evidence of benefit provides motivation to patients; opportunity for patients to connect with other patients receiving the intervention  Patient  ***Capability to participate:*** Support from family facilitates adherence to treatment; an intrinsic desire to engage with a model of care  ***Willingness to participate:*** To improve their health status; to improve the symptoms of their chronic condition |
| Rupasinghe | 2021 | Australia | Asthma | External context  ***Economic climate and governmental financing:*** Funding model incentivises short consultation times which limits the delivery of interventions that take time to deliver; insufficient funding for the intervention  ***Financial incentives:*** Lack of financial incentive for the practice to deliver the intervention  Organisation  ***Resources:*** Limited time to deliver the intervention due to existing busy workloads; lack of access to the equipment needed to deliver the intervention; cost of the equipment needed to deliver the intervention; lack of nurses trained to deliver the intervention; lack of appropriate private space to deliver the intervention  ***Involvement:*** Lack of teamwork  Professional  ***Professional role:*** Lack of confidence in nurse skills by GPs is a barrier to a greater role for nurses; perception by nurses that GPs do not value their role within the model of care  ***Competency:*** Insufficient training limits capacity to deliver the intervention  ***Philosophy of care:*** A conflict between the patient’s cultural identity and the intervention  Intervention  ***Nature and characteristics:*** The intervention is complicated to deliver  Patient  ***Relevance to self:*** A belief that their condition is low priority  ***Willingness to participate:*** Fear of social stigma | Organisation  ***Resources:*** Adequate staffing  Professional  ***Attitudes to change:*** An interest in the intervention  Intervention  ***Nature and characteristics:*** Linkage with practice electronic medical records software  ***Implementability:*** Training that is available locally |
| Sandlund | 2017 | Sweden | Insomnia |  | Intervention  ***Nature and characteristics:*** Patient resources that can be adapted to meet individual patient needs; nurse involvement in the development of resources enables the development of patient-centred resources |
| Shaw | 2013 | United States | Hypertension | External context  ***Infrastructure:*** Lack of centralised IT infrastructure to support ongoing delivery of the intervention  Professional  ***Attitudes to change:*** Reluctance by nurses to engage with the model of care due to uncertainty about the time commitment needed; competing demands of general practice; uncertainty about how to cover staff leave  Intervention  ***Nature and characteristics:*** Lack of access to intervention software by all staff; alert fatigue from software reminders; incompatibility of intervention software with existing IT systems  ***Implementability:*** Delays in implementation following training can result in a loss of knowledge about the intervention; delays in implementation can result in loss of time allocated to the intervention; delays in implementation can result in a loss in enthusiasm to implement the model of care | External context  ***Stakeholder buy-in:*** Agreement by administration that intervention is valuable; agreement by administration that the model of care fits within the values of the organisation  Organisation  ***Resources:*** Access to existing resources for use in the intervention  ***Relationship:*** Prior experience with research is a facilitator to practice engagement in new models of care  Professional  ***Attitudes to change:*** Previous experience in similar models of care enabled confidence in implementation; belief that the implementation of the model of care will be successful; models of care that enable nurses to have a broader scope of practice facilitate greater nurse engagement; models of care that enable nurses to have a broader scope of practice facilitate greater job satisfaction for nurses; experience delivering similar models of care that are considered valuable by practice staff  Intervention  ***Nature and characteristics:*** Perceived benefits of the model of care for patients facilitates engagement of health care professionals; alignment of the model of care with existing work practices; telephone-delivered interventions are convenient for patients; telephone-delivered interventions are cost effective; delivery of the intervention with other established interventions that are seen as valuable  ***Implementability:*** Minimal time needed for implementation can be a facilitator to maintaining enthusiasm in implementation. |
| Soejbjerg | 2024 | Denmark | Type 2 diabetes and ischaemic heart disease | Organisation  ***Resources:*** Limited time to deliver the intervention due to existing busy workloads; staff turnover increases workloads for remaining staff  Professional  ***Philosophy of care:*** Lack of patient-centred care is barrier to patient engagement; differing priorities for care between the patient and clinician; differing expectations of the role of the GP between the GP and patient  Patient  ***Willingness to participate:*** Awareness of the demands on GPs limits patient engagement due to concern about burdening the GP | Organisation  ***Relationship:*** A successful nurse-patient relationship improves patient engagement with the model of care; a pre-existing relationship with the patient is a facilitator to delivery of a model of care; a pre-existing relationship with the patient is a facilitator to determining suitability of an intervention  Intervention  ***Nature and characteristics:*** Structured protocols |
| Stenner | 2011 | UK | Diabetes |  | Organisation  ***Processes and systems:*** Continuity of care facilitates trust in the nurse-patient relationship; Longer appointment times facilitate patient engagement with models of care  ***Involvement:*** Evidence of teamwork between GPs and nurses instils confidence in patients  Professional  ***Philosophy of care:*** A person-centred approach to care is a facilitator to patient engagement; a holistic approach to care is a facilitator to patient engagement |
| Stephen | 2018 | Australia | Hypertension | Organisation  ***Resources:*** Limited time to deliver the intervention due to existing busy workloads  Intervention  ***Implementability:*** Implementing a new model of care adds work to existing workload | Organisation  ***Resources:*** Adequate staffing  ***Processes and systems:*** Longer appointment times facilitate patient engagement with models of care  ***Involvement:*** Evidence of teamwork between nurses and GPs instils confidence in patients; GP involvement in the model of care increases patients’ confidence in the intervention  Professional  ***Philosophy of care:*** A person-centred approach to care is a facilitator to patient engagement; an approachable communication style, without the use of technical language, enhances the nurse-patient relationship  Intervention  ***Nature and characteristics:*** Structured protocols; structured follow up care facilitates patient motivation |
| Stephen | 2024 | Australia | Hypertension | External context  ***Economic climate and governmental financing:*** Lack of funding for nurse-delivered care  Organisation  ***Resources:*** Limited time to deliver the intervention due to existing busy workloads  ***Processes and systems:*** Short appointments with GPs are a barrier to communication by the patient  ***Involvement:*** Lack of GP support for the implementation of the model of care; lack of support from the practice manager to implement the model of care  Patient  ***Capability to participate:*** Competing health priorities | External context  ***Economic climate and governmental financing: F***unding for nurse-delivered interventions  Organisation  ***Processes and systems:*** Longer appointment times facilitate patient engagement with models of care ; regular appointments provide accountability for patients to adhere to treatment; availability of appointments with nurses  ***Relationship:*** Effective communication between GPs and nurses  ***Skill mix:*** Defining the responsibilities of team members is a facilitator to collaborative care  ***Involvement:*** Evidence of teamwork between GPs and nurses instils confidence in patients; GP involvement in the model of care increases patients’ confidence in the intervention  Professional  ***Attitudes to change:*** Clinician awareness for a need for an intervention  ***Philosophy of care:*** A person-centred approach to care is facilitator to patient engagement  Patient  ***Willingness to participate:*** To improve their knowledge about their chronic condition; to improve their health status; a desire to make change |
| Turner | 2008 | The Netherlands | Coronary heart disease and heart failure |  | Intervention  ***Implementability:*** The delivery of an intervention by a trained specialist nurse is economically viable |
| Upton | 2011 | UK | Asthma | Organisation  ***Resources:*** Limited time to deliver the intervention due to existing busy workloads | Organisation  ***Relationship:*** Shared decision-making facilitates patient engagement with the intervention |
| van Bruggen | 2008 | The Netherlands | Type 2 diabetes | External context  ***Financial incentives:*** Lack of financial incentive for the practice to deliver the intervention  Organisation  ***Resources:*** Limited time to deliver the intervention due to existing busy workloads  Professional  ***Attitudes to change:*** Lack of motivation by practice staff; health care professional lack of confidence about the appropriateness of the intervention  ***Competency:*** Lack of knowledge about relevant guidelines | Professional  ***Competency:*** Formal training with supplemental practical on the job training improves engagement by general practice staff |
| van der Zweerde | 2020 | The Netherlands | Insomnia | Professional  ***Attitudes to change:*** Lack of mention of the intervention by GPs within consultations | Intervention  ***Nature and characteristics:*** Online-delivered interventions are time efficient |
| van Gaalen | 2016 | The Netherlands | Asthma | External context  ***Stakeholder buy-in:*** Lack of financial agreements with insurance companies for the funding of the intervention  Organisation  ***Processes and systems:*** Nurses working for practices without a structured approach to chronic disease management experience a lack of confidence in care delivery; lack of a structured approach to chronic disease management with adequate protocols  ***Involvement:*** Lack of collegial support is a barrier to implementation by nurses  Professional  ***Attitudes to change:*** Health care professional uncertainty about the benefit of the intervention is a barrier to delivery of the intervention by nurses and GPs; uncertainty about the cost-effectiveness of the model of care  ***Competency:*** Evidence of lack of competence in health professionals is a barrier to patient engagement with the model of care; insufficient training leads to lack of confidence in the delivery of the intervention  Intervention  ***Nature and characteristics:*** A time-consuming intervention is a barrier to patient engagement; lack of evidence for the intervention is a barrier to patient engagement; patient engagement with online interventions can be poor due to them being considered impersonal; lack of integration into practice electronic medical record software can result in reduced use by clinicians  ***Safety and data privacy:*** Lack of security is a barrier to use of intervention software by patients  Patient  ***Capability to participate:*** Illiteracy; advanced age; language barrier  ***Relevance to self:*** A belief that their condition is not serious enough to need the intervention  ***Willingness to participate:*** Non-attendance at appointments; non-adherence to treatment | External context  ***Infrastructure:*** Larger-sized practices enable greater support for nurses from colleagues  Organisation  ***Resources:*** Adequate staffing; sufficient equipment required to deliver the intervention  ***Skill mix:*** Defined roles for all team members  Intervention  ***Nature and characteristics:*** Patient resources that can be adapted to meet individual patient needs; a no cost intervention is preferred by patients; alignment of the model of care with existing work practices; opportunity for patients to connect with other patients receiving the intervention |
| Verwey | 2016 | The Netherlands | COPD and type 2 diabetes | Organisation  ***Resources:*** IT connectivity issues limit use of technology  ***Relationship:*** Delivering a new intervention to patients that nurses have known for a long time can be difficult  Professional:  ***Attitudes to change:*** Uncertainty about the appropriateness of the intervention for the patient group  ***Competence:*** Insufficient training leads to lack of confidence in the delivery of the intervention  Intervention  ***Nature and characteristics:*** Insufficient patient assessment within the model of care to adapt the intervention to patient need; use of unfamiliar terminology is a barrier for some patients  ***Implementability:*** Implementing a new model of care is time consuming | Intervention  ***Nature and characteristics:*** Easy to use resources; remote monitoring of patient progress facilitates nurse delivery of the intervention |
| Voncken-Brewster | 2014 | The Netherlands | COPD | Professional  ***Attitudes to change:*** A perception by health care professionals that the intervention only benefits a subgroup of patients  Intervention  ***Nature and characteristics:*** Patient resources that cannot be adapted to meet individual patient needs have reduced use by patients  Patient  ***Capability to participate:*** Lack of time to commit to the intervention; lack of access to the technology needed for the intervention  ***Relevance to self:*** A belief that they do not need the intervention; a belief that the intervention does not meet their needs | Organisation  ***Processes and systems:*** Sending prompts to patients  Intervention  ***Nature and characteristics:*** Capacity to generate succinct reports from software; linkage with practice electronic medical records software; provision of model of care-branded stationary to patients is a motivator for patient engagement; access to data related to patient progress provides motivation to nurses |
| Wald | 2004 | UK | Heart failure | Organisation  ***Resources:*** Limited time to deliver the intervention due to existing busy workloads | Organisation  ***Relationship***: An educational and clinical link between primary care and secondary care is a facilitator to the establishment of models of care |
| Walker | 2014 | New Zealand | Type 2 diabetes and hypertension |  | Organisation  ***Relationship:*** A working relationship between a practice nurse and a specialist nurse facilitates education of the practice to deliver evidence-based care |
| Walters | 2012 | Australia | COPD | External context  ***Economic climate and governmental financing:*** Insufficient funding for the intervention  Organisation  ***Resources:*** Limited time to deliver the intervention due to existing busy workloads  Professional  ***Attitudes to change:*** Telephone delivered interventions are not given the same level of priority as face-to-face interventions; competing demands of general practice  Intervention  ***Nature and characteristics:*** Inadequate patient resources for patients with limited literacy | Organisation  ***Involvement:*** GP support of the model of care increases patients’ confidence in the intervention  Intervention  ***Nature and characteristics:*** Frequent appointments support the delivery of self-management care to patients |
| Watson | 2018 | UK | Prostate cancer | Organisation  ***Skill mix:*** Insufficient opportunities to deliver the intervention can result in difficulty maintaining competency  Professional  ***Philosophy of care:*** Lack of engagement by patients due to a perceived lack of priority is a disincentive for nurse engagement |  |
| Weinberger | 1995 | United States | Type 2 diabetes |  | Intervention  ***Nature and characteristics:*** Telephone-delivered interventions work for case management delivered to large geographical populations |
| Weise | 2023 | Germany | Multiple conditions: type 2 diabetes, hypertension | Professional  ***Professional role:*** Lack of clarity about the scope of practice of nurses is a barrier to patient engagement  ***Competence:*** Lack of confidence in the competence of the nurse is a barrier to patient engagement; concern by patients that nurses could work beyond their scope of practice; uncertainty about the appropriateness of nurse-delivered care is a barrier to patient engagement  Patient  ***Relevance to self:*** Uncertainty about the benefits of the model of care | External context  ***Economic climate and governmental financing:*** Nurse-delivered care is cheaper than GP-delivered care  Organisation  ***Processes and systems:*** An organised practice is a facilitator to patient engagement  ***Relationship:*** Trust is a facilitator to patient engagement; regular contact with the GP is a facilitator to patient engagement; respect and trust between team members  ***Involvement:*** Supervision of the nurse by the GP is a facilitator to patient engagement  Professional  ***Philosophy of care:*** A nursing approach to communication is a facilitator to patient satisfaction; delivery of care by nurses is a facilitator to patient engagement with treatment  ***Competence:*** Evidence of a nurse’s qualification is a facilitator to patient engagement  Intervention  ***Nature and characteristics:*** Use of structured protocols is a facilitator to patient confidence in nurse delivery of care; development of protocols by GPs is a facilitator to patient engagement  Patient  ***Relevance to self:*** A belief that they would receive faster treatment  ***Willingness to participate:*** To support a greater role for general practice nurses |
| Weldam | 2017 | The Netherlands | COPD | External context  ***Economic climate and governmental financing:*** Lack of funding for nurse-delivered care  Professional  ***Attitudes to change:*** Insufficient knowledge about the intervention before deciding on involvement  Intervention  ***Nature and characteristics:*** Appointment schedule does not align with existing work practices; requirement for the GP to authorise the nurse to deliver the intervention  Patient  ***Capability to participate:*** Language barrier; multiple health issues; low socio-economic status; lack of access to the resources needed to participate in the intervention; limited health literacy | Organisation  ***Processes and systems:*** Compatibility of the intervention with existing work practices  Professional  ***Competency:*** Sufficient training enables confidence in the delivery of the intervention  Intervention  ***Nature and characteristics:*** Models of care that add value to the role of the nurse; models of care that facilitate the delivery of patient-centred care by nurses; easy to use resources; structured protocols; evidence of benefits to patients; alignment of the model of care with the underlying philosophy of nursing care; linkage with practice electronic medical records software |
| Wiener-Ogilvie | 2008 | UK | Asthma | Organisation  ***Resources:*** Limited time to deliver the intervention due to existing busy workloads; lack of time in appointments means other urgent clinical issues take priority; Lack of time for team meetings to discuss implementation; implementing new interventions increases workloads  ***Culture:*** General practice is an environment of constant change  Professional  ***Professional role:*** Insufficient nurse knowledge and skills reduces GP confidence in delegating responsibilities to nurses  ***Skill mix:*** Delegation of responsibilities to the nurse can result in a loss of GP knowledge; lack of clarity about team members roles; inconsistent approach to delivery of the intervention by team members  ***Competency:*** Updating knowledge to deliver interventions increases workloads  ***Attitudes to change:*** Health care professional lack of confidence about the appropriateness of the intervention  ***Philosophy of care:*** Lack of accountability for self-management by patients makes delivery of the intervention difficult  Intervention  ***Nature and characteristics:*** Irrelevance of the model of care to the clinical need in appointments  Patient  ***Capability to participate:*** Lack of confidence in their role in the intervention  ***Willingness to participate:*** Fear of the intervention; non-attendance at appointments; lack of motivation | Organisation  ***Culture:*** Positive workplace culture  ***Skill mix:*** Delegation of all responsibilities to the nurse facilitates a consistent approach to delivery of the intervention; shared responsibilities between team members  ***Relationship:*** Respect and trust between team members  Professional  ***Professional role:*** Sufficient nurse knowledge and skills is a facilitator to delegation of responsibilities by GPs; effective nurse communication skills  ***Attitudes to change:*** Confidence in the model of care improves health care professional engagement  Intervention  ***Nature and characteristics:*** A model of care that facilitates an empathetic approach to care facilitates health care professional engagement with a model of care |
| Wolf | 2013 | USA | Type 2 diabetes | Patient  ***Capability to participate:*** Low literacy |  |
| Woodcock | 1999 | UK | Type 2 diabetes | Organisation  ***Resources:*** Limited time to deliver the intervention due to existing busy workloads  Professional  ***Attitudes to change:*** Clinician uncertainty of usability of patient resources | Professional  ***Competency:***  Ongoing training support facilitates continued engagement by nurses |
| Wright | 2001 | UK | Ischaemic heart disease | Professional  ***Attitudes to change:*** Lack of interest by nurses in extending their scope of practice to deliver new models of care  ***Competency:*** Lack of knowledge about the chronic condition reduces the quality of care provided; Insufficient training leads to lack of confidence in the delivery of the intervention  ***Philosophy of care:*** Patient reluctance to change habits despite education from the nurse  Patient  ***Relevance to self:*** A belief that their condition is untreatable | Organisation  ***Processes and systems:*** Longer appointment times facilitate the development of the nurse-patient relationship  ***Involvement:*** Effective teamwork within the practice  Professional  ***Professional role:*** Confidence in nurses to seek support from GPs  ***Competency:*** Ongoing training facilitates confidence in the delivery of interventions  ***Philosophy of care:*** An empathetic approach by nurses enhances the nurse-patient relationship  Patient  ***Relevance to self:*** Patients that are managing well with their condition |
| Zwar | 2022 | Australia | Asthma | Organisation  ***Skill mix:*** Staffing changes during the delivery of the intervention  Professional  ***Attitudes to change:*** The demand of the model of care on the role of the nurse limits engagement by practices |  |

| Table 3: Legend  Primary themes  ***Secondary themes*** |
| --- |
